# Network analysis of relationships among psychopathology, cognitive function, and psychosocial functioning in independent samples of Chinese with schizophrenia or bipolar disorder

**DOI:** 10.1017/S0033291725102481

**Published:** 2025-11-20

**Authors:** Hua Yu, Weiyan Wang, Mengxuan Qiao, Min Yang, Xiaojing Li, Wei Wei, Yamin Zhang, Mingli Li, Qaing Wang, Wei Deng, Wanjun Guo, Tao Li

**Affiliations:** 1 Translational Psychiatry, Hangzhou No. 7 People’s Hospital, Zhejiang University School of Medicine, Hangzhou, China; 2 Affiliated Mental Health Center, Zhejiang University School of Medicine, Hangzhou, China; 3 Mental Health Center, West China Hospital of Sichuan University, Chengdu, China

**Keywords:** cognitive function, directed acyclic graph, network analysis, psychopathology, severe mental illness, schizophrenia, bipolar disorder, disorganized symptoms, depressive symptoms, psychosocial functioning, processing speed, transdiagnostic

## Abstract

**Background:**

How psychotic symptoms, depressive symptoms, cognitive deficits, and functional impairment may interact with one another in schizophrenia or bipolar disorder is unclear.

**Methods:**

This study explored these interactions in a discovery sample of 339 Chinese, of whom 146 had first-episode schizophrenia and 193 had bipolar disorder. Psychotic symptoms were assessed using the Positive and Negative Symptom Scale; depressive symptoms, using the Hamilton Depression Rating Scale; cognitive deficits, using tests of processing speed, executive function, and logical memory; and functional impairment, using clinical assessments. Network models connecting the four types of variables were developed and compared between men and women and between disorders. Potential causal relationships among the variables were explored through directed acyclic graphing. The results in the discovery sample were compared to those obtained for a validation sample of 235 Chinese, of whom 138 had chronic schizophrenia and 97 had bipolar disorder.

**Results:**

In the discovery and validation cohorts, schizophrenia and bipolar disorder showed similar networks of associations, in which the central hubs included ‘disorganized’ symptoms, depressive symptoms, and deficits in processing speed during the digital symbol substitution test. Directed acyclic graphing suggested that disorganized symptoms were upstream drivers of cognitive impairment and functional decline, while core depressive symptoms (e.g. low mood) drove somatic and anxiety symptoms.

**Conclusions:**

Our study advocates for transdiagnostic, network-informed strategies prioritizing the mitigation of disorganization and depressive symptoms to disrupt symptom cascades and improve functional outcomes in schizophrenia and bipolar disorder.

## Introduction

Schizophrenia and bipolar disorder are two severe mental health disorders that exert substantial impacts on the affected individuals, their caregivers, and the larger society (Merikangas et al., 2011; Saha, Chant, & McGrath, 2007). They overlap substantially in psychopathology and cognitive deficits (Chavez-Baldini et al., 2023), including deficits in executive function, working memory, and processing speed (Martinez-Aran & Vieta, 2015), which can persist even when other clinical symptoms have improved (Fett et al., 2020; MacQueen & Memedovich, 2017). Both disorders involve profound functional impairments that critically disrupt an individual’s ability to fulfill societal roles, maintain occupational engagement, and participate meaningfully in daily life (Bonnín et al., 2019; Kalisova et al., 2023). Schizophrenia manifests primarily as psychotic symptoms, but depressive symptoms often co-occur or appear later (Upthegrove, Marwaha, & Birchwood, 2017). Bipolar disorder manifests primarily as depressive and (hypo)manic symptoms, but psychotic symptoms are sometimes present (Perlis, Brown, Baker, & Nierenberg, 2006). ‘Dimensional’ approaches consider the two disorders as a single entity differing in dimensions and severity (Shao, Simmonds-Buckley, Zavlis, & Bentall, 2024), even if their diagnostic criteria differ according to the Diagnostic and Statistical Manual of Mental Disorders and the International Classification of Diseases.

Clarifying the relationship among clinical psychopathology, cognitive function and occupational functioning between these two disorders is important for understanding their onset, progression and treatment. For example, understanding whether and how the symptoms of the disorders interact and which ones occur earlier than others in each disorder could help improve and personalize treatments (Izquierdo et al., 2021). This is especially important given that current psychological and pharmacological interventions for these disorders often still prove ineffective against functional impairments in many individuals with schizophrenia and bipolar disorders (Martinez-Aran et al., 2007; Sheffield, Karcher, & Barch, 2018).

Network analysis may be able to shed light on these questions because it treats mental disorders as networks of interconnected symptoms rather than as discrete entities causing symptoms (Moroń et al., 2025; Qiao et al., 2024). Network analysis can reveal, for example, that some symptoms are more interconnected and central than others and may therefore give rise to the others (Jiménez, de Montis, & Garza-Villarreal, 2025). By quantifying the strength of connections among the symptoms, the network can provide insights into disorder severity and progression (Robinaugh, Millner, & McNally, 2016). In these ways, network analysis may be able to prioritize which symptoms are core to a disorder and should be treated first. As a valuable complement to network analysis, directed acyclic graphing (DAG) can further investigate the relationships among variables to impute causal connections (Lipsky & Greenland, 2022).

While network analysis excels at mapping symptom co-occurrences and identifying central nodes, it often falls short of establishing definitive causal pathways without longitudinal or interventional data. In contrast, the DAG approaches leverage probabilistic reasoning to infer directional and conditional dependencies, thereby offering a more rigorous framework for causal inference (Limongi et al., 2023; Silva et al., 2023). Thus, where network analysis identifies which symptoms are central, DAGs elucidate how and why they are causally implicated in the disorder’s trajectory (Mackinley et al., 2023).

To our knowledge, network analysis has yet to be applied cross-diagnostically to schizophrenia and bipolar disorder while taking into account psychotic symptoms, mood symptoms, cognitive function, and personal functioning. The present study aimed to do this in two independent samples of Chinese to ensure robust and valid findings.

## Methods

### Participants

The discovery cohort comprised individuals receiving psychiatric care at the Mental Health Center of West China Hospital, Sichuan University (Chengdu, China): 146 individuals (86 females) with first-episode schizophrenia who had received antipsychotic medication for 0–3 days by the time of study enrollment and 193 individuals (108 females) with bipolar disorder of depressive (hypo) manic or euthymic types who were on various pharmacotherapy regimes at enrollment. Diagnoses were made independently by two licensed psychiatrists using the Structured Clinical Interview for Axis I Disorders from the 4th edition of the *Diagnostic and Statistical Manual of Mental Disorders*.

The validation cohort comprised individuals from both inpatient and outpatient psychiatric facilities at Hangzhou No. 7th People’s Hospital (Hangzhou, China): 138 individuals (44 females) with chronic schizophrenia and 97 individuals (27 females) with bipolar disorder of depressive, (hypo) manic or euthymic types. Diagnoses were made according to the fifth edition of the *Diagnostic and Statistical Manual of Mental Disorders.* These individuals were on various pharmacotherapy regimes at enrollment.

Individuals were excluded from the cohorts if they reported substance or alcohol abuse during the previous year, or if they had severe medical or neurological comorbidities. All participants were right-handed, and they, or their legal representatives, provided written informed consent. The study protocol was approved by the Ethics Committees at both study sites before recruitment, which ran from July 2013 to December 2024.

### Assessments of psychopathological symptoms

Psychotic symptoms were assessed with all participants using the 30-item Positive and Negative Symptom Scale (PANSS), which assesses the severity of psychotic symptoms in terms of five factors: positive, negative, disorganized/concrete, excited, and depressed (Wallwork et al., 2012). This five-factor model is superior to a three-factor model for assessing psychotic symptoms (Wallwork et al., 2012).

Depressive symptoms in individuals with both disorders were assessed on the Hamilton Depression Rating Scale (HAMD) in terms of subscores for the following four factors (Shafer, 2006): anxiety (items 9, 10, 11, 15, 17), depression (items 1, 2, 3, 7, 8), insomnia/sleep difficulty (items 4, 5, 6), and somatic symptoms (items 12, 13, 14, 16).

Manic symptoms were assessed in individuals with bipolar disorder using the Young Mania Rating Scale (YMRS), a clinician-administered tool of 11 items that collects data on elevated mood, increased motor activity energy, irritability, speech rate and volume, thought content, disruptive–aggressive behavior, and sleep patterns. The total score ranges from 0 to 60, with a cutoff of ≥12 typically indicating clinically relevant mania (Young, Biggs, Ziegler, & Meyer, 1978).

All individuals were subjected to a battery of cognitive tests: processing speed was assessed in the digital symbol substitution test and part A of the trail-making test, with reverse scoring for time to completion; executive function was assessed in part B of the trail-making test, with reverse scoring for time to completion; and logical memory was assessed through immediate and delayed recall tasks (Saleh et al., 2017).

## Assessments of functioning

Psychological, social, and occupational functioning were assessed in all participants in the discovery cohort using the global assessment of functioning (GAF), which has been widely used (Monrad Aas, 2014). Scores are categorized into 10-point ranges, such that scores of 1–10 indicate severe self-harm risk, while scores of 91–100 indicate optimal functioning (Picco et al., 2018). Surveys were filled out during clinical interviews, which were initially conducted by two raters. An experienced investigator resolved discrepancies exceeding 10 points. Once inter-rater consistency remained within 10-point intervals, subsequent clinical interviews were conducted by a single rater to ensure reliability.

Psychosocial functioning in the validation sample was assessed using the Personal and Social Performance Scale (PSP) (Tianmei et al., 2011), which evaluates four domains: socially useful activities (e.g. work/study), personal/social relationships, self-care, and disruptive/aggressive behaviors (Tsouvalas et al., 2011). Scores are categorized into three levels: 71–100 (mild impairment), 31–70 (disability), and 0–30 (severe dysfunction requiring intensive support). This instrument was chosen for the validation sample instead of the GAF because of its superior face validity and psychometric reliability (Morosini et al., 2000).

## Data processing

All statistical analyses were conducted using SPSS 29.0 and R 4.0.3 (https://www.r-project.org/). Missing values were visualized using the ‘vim’ package in R, and missing data were substituted through multiple imputation (Zhang, 2016). We considered this approach appropriate under the assumption that data were missing randomly without bias. If data for a given variable were missing for >20% of either cohort, the variable was not included in the final analysis.

Clinical characteristics, symptom severity, and functioning were compared between males and females and between those with schizophrenia or bipolar disorder. Differences in continuous variables were assessed for significance using independent-samples tests, while differences in categorical variables were assessed using the chi-squared test. Differences were considered significant if associated with p < 0.05.

### Network analyses

Potential relationships among psychosocial functioning, cognitive impairment, affective symptoms, and psychotic manifestations were explored through network analyses using the packages ‘bootnet’, ‘qgraph,’ and ‘mgm’ in R (Huang et al., 2024). Nodes in the network represent clinical or cognitive constructs, while edges represent regularized partial correlations that take into account covariates. Key nodes were identified based on the centrality indices of strength, closeness, betweenness, and expected influence (Batool & Niazi, 2014). Network models were developed using the enhanced LASSO algorithm based on logistic regression and regularization penalties (Epskamp, Borsboom, & Fried, 2018). Excessive model complexity was penalized using the extended Bayesian information criterion. Stability and accuracy of edge weights and centrality metrics were assessed through non-parametric bootstrapping (2,500 iterations) and case-dropping bootstrapping of stability coefficients. Node predictability was quantified based on the proportion of observed variance that could be explained using a pairwise mixed graphical model. Network analyses were conducted at syndrome-level resolution based on 17 nodes: positive, negative, disorganized, excited, or depressed factors on the PANSS; anxiety, depression, insomnia or somatic factors on the HAMD; YMRS score (mania); personal functioning; cognitive domain (TMT-A/B time, digital symbol substitution test, immediate/delayed logical memory test); and age at disorder onset. Network analyses were also conducted at item-level resolution based on 45 nodes (i.e. items of the HAMD and part of the PANSS items, cognition, personal functioning and duration of illness).

Directed acyclic graphing was performed as described (Huang et al., 2024) to investigate causal pathways among cognitive function, psychopathological symptoms, and personal functioning. Such graphing can disentangle direct from indirect effects in multivariate systems (McNally, Heeren, & Robinaugh, 2017).

Network structures were compared between males and females, as well as between schizophrenia and bipolar disorder, using the network comparison test in R (Sun et al., 2025), which compared the invariance of network structure, edge strength and global strength between networks. Comparisons were performed using null distributions based on 5,000 permutations, with α = 0.05. Results from edge-level tests were corrected for multiple comparisons using the Holm–Bonferroni method (van Borkulo et al., 2023).

## Results

### Characteristics of both samples

In the discovery sample, males showed significantly longer times on part B of the trail-making test and significantly worse performance on the digital symbol substitution test (Table 1). Males and females did not differ significantly in age, years of education, scores on the GAF, or subscores on the HAMD or PANSS.

**Table 1.** Descriptive summary of the participants and gender group effects

	Total	Male	Female	χ^2^/t	P	φc/Cohen’s *d*
Age	23.99 (8.05)	24.19 (7.79)	23.83 (8.26)	−0.41	0.69	0.045
Education years	12.86 (2.99)	12.79 (3.05)	12.91 (2.95)	0.37	0.71	−0.04
Duration of illness (months)	34.84 (43.69)	36.73 (46.72)	33.35 (41.27)	−0.67	0.50	0.077
Age of onset (years)	20.89 (7.31)	20.60 (6.24)	21.12 (8.05)	0.64	0.53	−0.072
**Diagnosis**				0.30	0.59	−0.029
SCZ	146	60	86			
BD	193	85	108			
**PANSS**						
Positive factor	9.82 (5.45)	9.56 (5.31)	10.03 (5.56)	0.77	0.44	−0.086
Negative factor	14.09 (7.84)	14.36 (8.07)	13.89 (7.68)	−0.54	0.59	0.06
Disorganized factor	7.30 (3.79)	7.66 (4.04)	7.02 (3.57)	−1.51	0.13	0.17
Excited factor	7.78 (4.18)	7.59 (4.26)	7.93 (4.11)	0.74	0.46	−0.081
Depressed factor	7.02 (3.08)	6.80 (2.93)	7.18 (3.19)	−1.13	0.26	−0.13
**HAMD**						
Anxiety	2.73 (2.31)	2.78 (2.29)	2.70 (2.33)	−0.31	0.76	0.035
Depression	4.22 (3.60)	3.84 (3.36)	4.49 (3.74)	1.66	0.098	−0.18
Insomnia	1.57 (1.62)	1.44 (1.61)	1.67 (1.63)	1.29	0.20	−0.14
Somatic symptoms	1.04 (1.41)	0.88 (1.28)	1.16 (1.49)	1.80	0.073	−0.20
**YMRS**	7.65 (8.30)	7.03 (8.10)	8.13 (8.43)	1.20	0.23	−0.13
**Cognitive function**						
TMT-A time	41.10 (17.03)	41.36 (17.50)	40.91 (16.72)	−0.23	0.82	0.026
TMT-B time	69.20 (32.10)	73.78 (36.02)	65.83 (28.51)	−2.17	**0.030**	0.25
DSB	50.80 (17.64)	48.31 (17.31)	52.62 (17.70)	2.12	**0.035**	−0.25
Logical memory immediately	7.69 (4.35)	7.17 (4.00)	8.08 (4.57)	1.84	0.067	−0.21
Logical memory delayed	5.88 (4.21)	5.45 (3.50)	6.18 (4.62)	1.46	0.15	−0.18
**GAF**	53.24 (14.30)	53.79 (14.42)	52.81 (14.24)	−0.62	0.53	0.068

*Note*: *P* < .05 are shown in bold. SCZ, schizophrenia; BD, bipolar disorder; PANSS, the Positive and Negative Symptom Scale; HAMD, Hamilton depression scale; YMRS, Young mania rating scale; TMT, trail making test; DSB, digital symbol substitution; GAF, global assessment of functioning.

When both sexes in the discovery sample were aggregated, individuals with schizophrenia were found to be significantly younger and less educated than those with bipolar disorder, and they had had their disorder for a shorter time (Table 2). Conversely, those with schizophrenia were found to have stronger positive symptoms, negative symptoms, disorganized thinking, and excitement. Individuals with bipolar disorder, for their part, showed more severe depressive symptoms, insomnia symptoms, and somatic symptoms on the HAMD.

**Table 2.** Comparison of demographic and clinical information among the diagnostic groups

	Total	SCZ	BD	χ^2^/t	P	φc/Cohen’s *d*
Age	23.99 (8.05)	22.25 (7.21)	25.26 (8.43)	−3.46	**<0.001**	−0.38
Education years	12.86 (2.99)	11.81 (3.00)	13.64 (2.74)	−5.85	**<0.001**	−0.64
Duration of illness (months)	34.84 (43.69)	16.22 (21.81)	48.57 (50.27)	−6.84	**<0.001**	−0.84
Age of onset (years)	20.89 (7.31)	20.96 (7.08)	20.84 (7.50)	0.15	0.88	0.016
Diagnosis				0.30	0.59	−0.029
Male	145	60	85			
Female	194	86	108			
**PANSS**						
Positive factor	9.82 (5.45)	14.14 (4.10)	6.40 (3.67)	18.01	**<0.001**	1.99
Negative factor	14.09 (7.84)	19.12 (7.46)	10.11 (5.52)	12.57	**<0.001**	1.37
Disorganized factor	7.30 (3.79)	9.13 (3.86)	5.85 (3.03)	8.63	**<0.001**	0.95
Excited factor	7.78 (4.18)	8.73 (4.38)	7.03 (3.86)	3.74	**<0.001**	0.41
Depressed factor	7.02 (3.08)	6.88 (2.96)	7.12 (3.18)	−0.69	0.49	−0.078
**HAMD**						
Anxiety	2.73 (2.31)	2.69 (2.31)	2.77 (2.31)	−0.30	0.77	−0.035
Depression	4.22 (3.60)	3.56 (2.93)	4.71 (3.96)	−2.95	**0.003**	−0.33
Insomnia	1.57 (1.62)	1.32 (1.48)	1.76 (1.71)	−2.49	**0.013**	−0.28
Somatic symptoms	1.04 (1.41)	0.77 (1.24)	1.24 (1.50)	−3.08	**0.002**	−0.34
**YMRS**	7.65 (8.30)	7.08 (6.79)	8.08 (9.24)	−1.09	0.28	−0.12
**Cognitive function**						
TMT-A time	41.10 (17.03)	41.58 (17.45)	40.73 (16.74)	0.44	0.66	0.05
TMT-B time	69.20 (32.10)	72.28 (34.55)	66.87 (30.00)	1.48	0.14	0.17
DSB	50.80 (17.64)	44.60 (15.74)	55.08 (17.65)	−5.34	**<0.001**	−0.63
Logical memory immediately	7.69 (4.35)	6.57 (4.11)	8.08 (4.57)	−4.07	**<0.001**	−0.35
Logical memory delayed	5.88 (4.21)	4.76 (3.76)	6.71 (4.34)	−4.03	**<0.001**	−0.48
**GAF**	53.24 (14.30)	48.11 (13.06)	57.08 (14.02)	−5.97	**<0.001**	−0.66

*Note*: *P* < .05 are shown in bold. SCZ, schizophrenia; BD, bipolar disorder; PANSS, the Positive and Negative Symptom Scale; HAMD, Hamilton depression scale; YMRS, Young mania rating scale; TMT, trail making test; DSB, digital symbol substitution; GAF, global assessment of functioning.

Compared to individuals with schizophrenia, those with bipolar disorder showed significantly better global functioning and significantly better digital symbol substitution and logical memory. The two groups did not differ significantly in performance on either part A or B of the trail-making test.

The demographic and clinical characteristics of the discovery cohort, and the differences between males and females in that cohort, were similar to those of the validation cohort (Supplementary Table S1). Males were significantly older with later illness onset. Females exhibited higher severity in PANSS negative and depressed factors, as well as elevated HAMD anxiety and depression scores. Males scored higher on YMRS. No significant sex differences were observed in education, PANSS positive/disorganized/excited factors, cognitive performance (TMT-A/B, DSB, logical memory), or psychosocial functioning (PSP).

The differences between the two diagnostic groups in the discovery cohort were also similar to those in the validation cohort (Supplementary Table S2). In the validation cohort, SCZ patients exhibited significantly higher PANSS positive, negative, and disorganized factor scores, reflecting core psychotic symptoms, while BD patients showed elevated YMRS and HAMD anxiety /insomnia scores. Cognitive and psychosocial performance differed marginally, with BD patients outperforming SCZ in DSB. No group differences were observed in age, education, illness duration, age of onset, TMT-A/B, or logical memory. Diagnoses showed balanced sex distribution.

### Network analysis at the syndrome level

**Figure 1.**
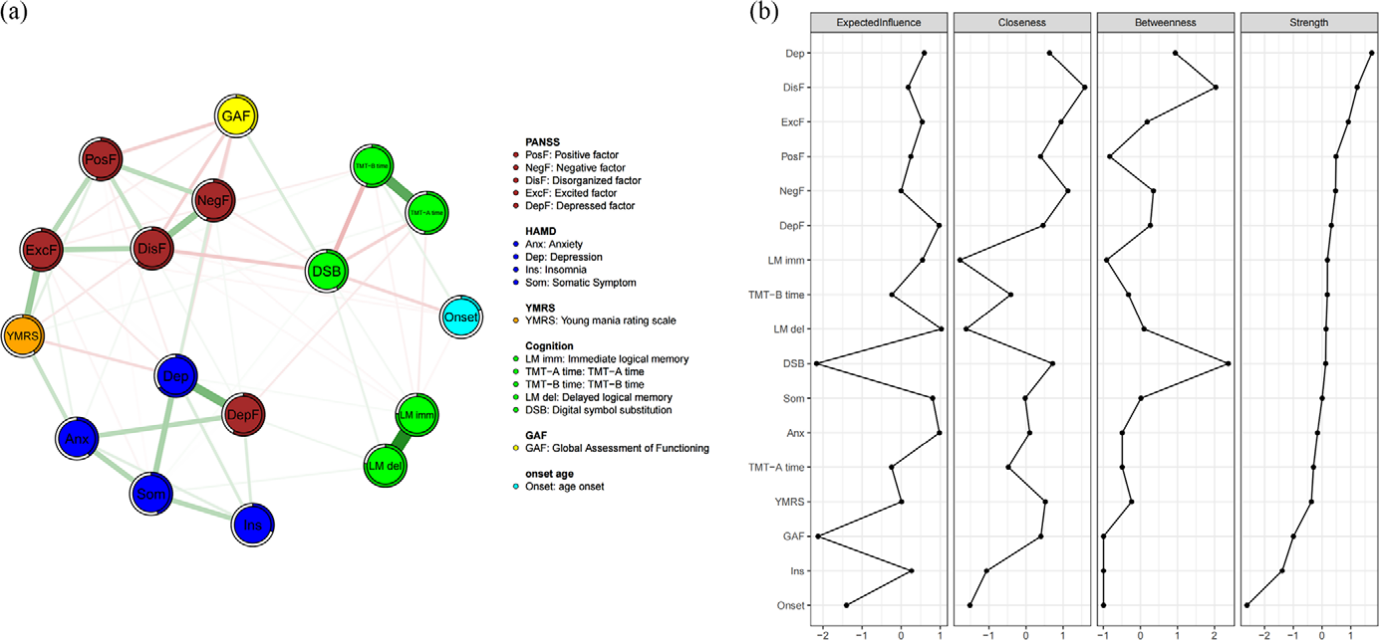
(a) The estimated regularized network structure of psychotic symptoms dimensions, mood symptom dimensions, cognitive dimensions, global assessment functioning and duration of illness in the trans-diagnostic sample (left) and (b) the centrality indices of nodes in the network (right). The value of each edge represents the strength of the correlations. The green edges (for the online version) or positive edge values (for the print version) indicate positive partial correlations, while the red edges (for the online version) or negative edge values (for the print version) indicate negative partial correlations. Thicker lines represent stronger connections. The ring around each node represents its predictability values. Centrality indices are shown as standardized z scores. *Note*: PosF, ‘positive factor’; NegF, ‘negative factor’; DisF, ‘disorganized factor’; ExcF, ‘excited factor’; DepF, ‘depressive factor’; Anx, ‘anxiety’; Dep, ‘depression’; Som, ‘somatic symptom’; Ins, ‘insomnia’; YMRS, ‘Young’s mania rating scale’; LM, ‘logical memory’; TMT, ‘trial making task’; DSB, ‘digital number substitution’; GAF, ‘global assessment of functioning’.

Network analysis of the discovery cohort revealed a complex interplay among psychotic symptoms, mood symptoms, cognitive function and personal functioning. Four clusters were observed (Figure 1a): *depression-related symptoms*, based on HAMD items related to depression, anxiety, insomnia, and somatic symptoms, as well as the depressive factor on the PANSS; *psychotic and excitatory symptoms*, based on the positive, negative and disorganized factors on the PANSS as well as total score on the YMRS; *cognition*, based on logical memory delay, time on parts A and B of the trail-making test, and performance on the digital symbol substitution test; and *personal functioning*, based on the GAF score.

We found that most within-cluster edges between psychopathology symptoms and most within-cluster edges between cognitive domains were positive, except for the connections between time on parts A and B of the trail-making test and other cognitive domains. Most cross-cluster edges between cognitive domains and psychopathology were negative, such that more severe symptoms were associated with worse cognitive function. Score on the GAF was associated negatively with severity of psychotic symptoms but positively with performance on the digital symbol substitution test.

The strongest nodes were the depression factor on the HAMD as well as the disorganized, excited and positive factors on the PANSS (Figure 1b). Performance on the digital symbol substitution test showed the most negative expected influence and betweenness, suggesting its role as a potential inhibitory bridge (Supplementary Table S3). Other cognitive variables generally showed moderate centrality but higher closeness. Most of the edges in the network were stable because they were robust (clustering coefficient = 0.75), and the confidence intervals of edge weights were fairly narrow.

Similar results were observed in the network analysis of the validation cohort (Supplementary Table S4, Supplementary Figure S1). In the validation cohort, four distinct clusters emerged, including depression-related symptoms, psychotic/excitatory symptoms, cognitive cluster and occupational performance. We found PSP’s negative association with psychotic symptoms and positive association with DSB. The strongest nodal influences were HAMD Depression and PANSS Disorganized factor, while DSB demonstrated critical inhibitory bridge functions through both the highest betweenness centrality and the most negative expected influence.

### Network analysis at the item level

**Figure 2.**
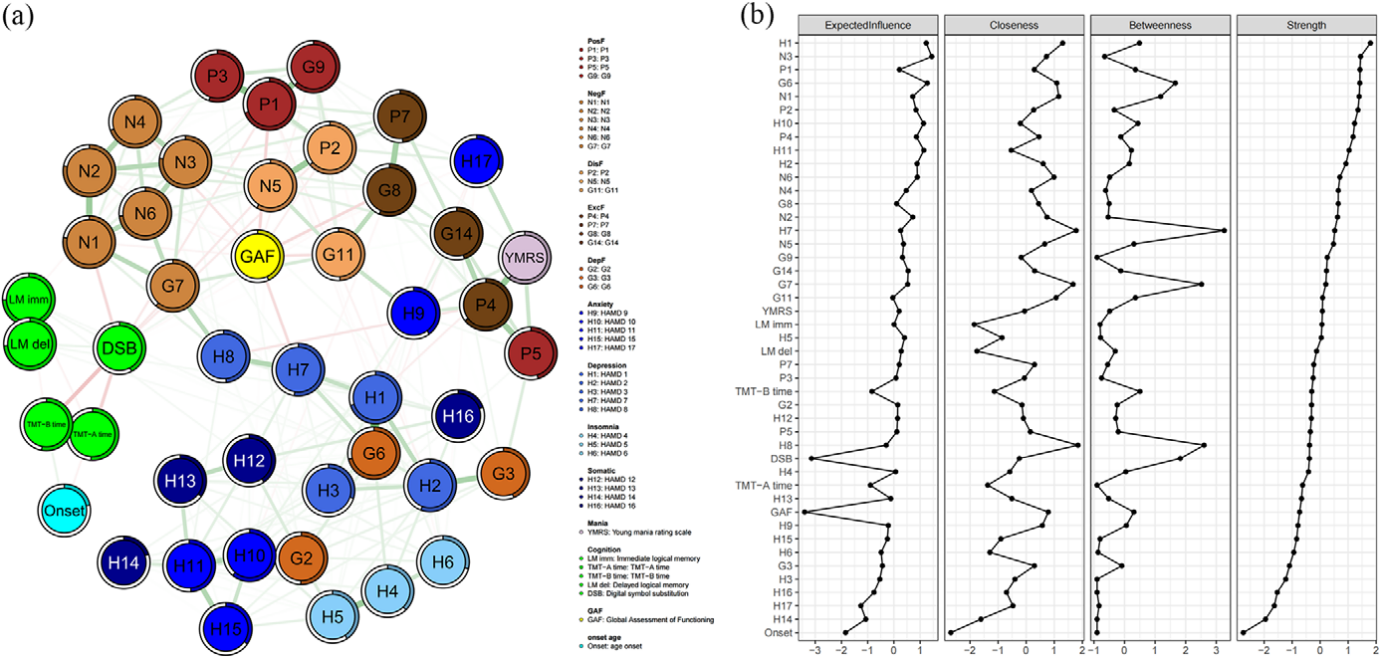
(a) The estimated regularized network structure of psychotic symptoms (item level), manic symptom, depressive symptom (item level), cognitive function, global assessment functioning and duration of illness in the transdiagnostic sample (left), and (b) the centrality indices of nodes in the network (right). The value of each edge represents the strength of the correlations. The green edges (for the online version) or positive edge values (for the print version) indicate positive partial correlations, while the red edges (for the online version) or negative edge values (for the print version) indicate negative partial correlations. Thicker lines represent stronger connections. The ring around each node represents its predictability values. Centrality indices are shown as standardized z scores. *Note*: P, ‘positive symptom’; N, ‘negative symptom’; G, ‘general psychopathology’; H, ‘Hamilton depression scale’; YMRS, ‘Young’s mania rating scale’; LM, ‘logical memory’; TMT, ‘trial making task’; DSB, ‘digital number substitution’; GAF, ‘global assessment of functioning’.

In network analysis of the discovery sample based on 45 nodes, specific psychopathological dimensions showed similar connections to cognitive function and personal functioning (Figure 2). Based on centrality indices, PANSS items N3 (poor rapport) and G6 (depression), as well as HAMD item H1 (depressed mood), emerged as central hubs, with high expected influence (Figure 2b, Supplementary Table S5). Based on betweenness, HAMD item 7 (work and activities) and PANSS item G7 (motor retardation) emerged as critical bridges. PANSS items N3 and HAMD item 1 (depressed mood) exerted the strongest positive influences (with high positive expected influence), while performance on the digital symbol substitution test and score on the GAF showed the strongest inhibitory effects (with negative expected influence). Negative symptoms on the PANSS and depression items on the HAMD emerged as structural linchpins, while performance on the digital symbol substitution test and other functional measures showed inhibitory roles and variable explanatory power.

These results for the discovery cohort were assessed to be stable and reliable (Supplementary Table S6). The overall results were similar for the validation cohort (Supplementary Figure S2).

### Directed acyclic graphing of causal relationships

**Figure 3.**
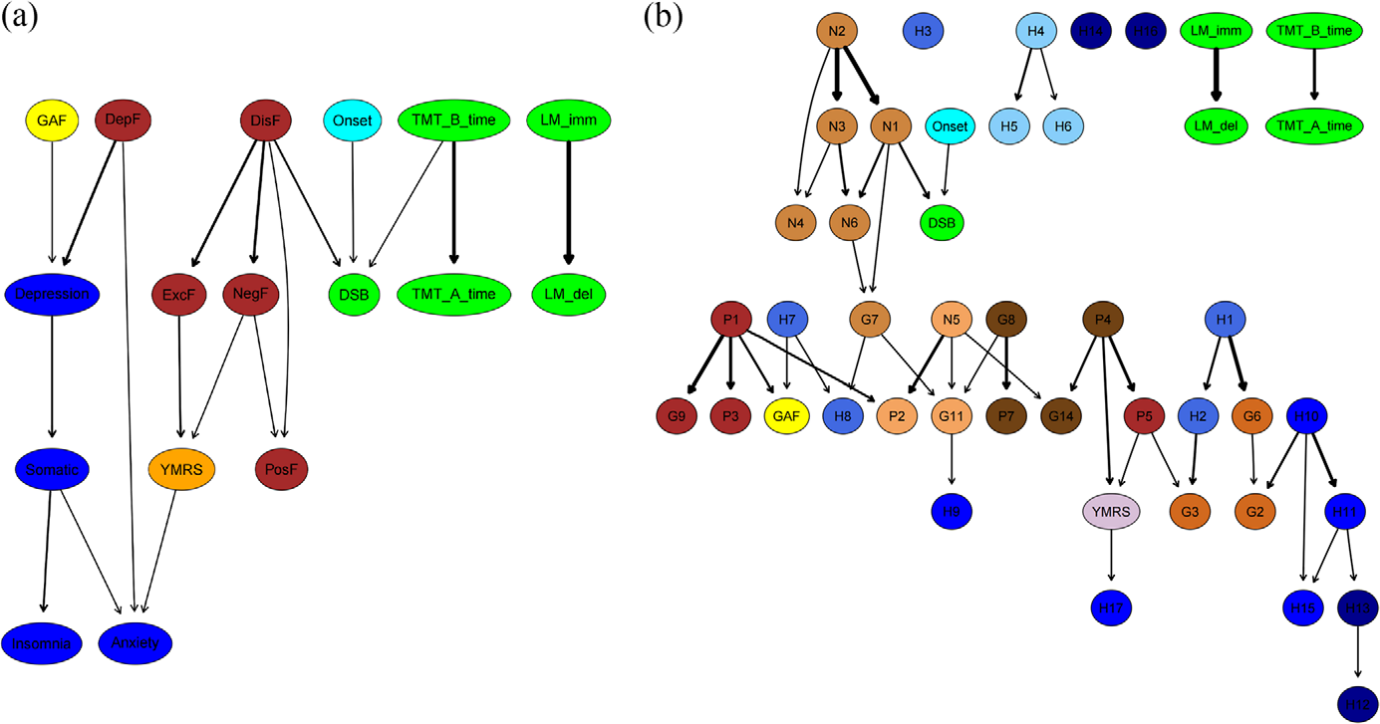
(a) A consensus Bayesian network (directed acyclic graph, DAG) depicting the associations among psychopathology, cognitive function, personal functioning, and illness duration at dimension-level in the discovery cohort. (b) A consensus Bayesian network (directed acyclic graph, DAG) depicting the associations among psychopathology, cognitive function, personal functioning and illness duration at item-level in the discovery cohort. Arrowheads show possibly predictive direction, with thicker lines for higher BIC values. *Note*: PosF, ‘positive factor’; NegF, ‘negative factor’; DisF, ‘disorganized factor’; ExcF, ‘excited factor’; DepF, ‘depressive factor’; Anx, ‘anxiety’; Dep, ‘depression’; Som, ‘somatic symptom’; Ins, ‘insomnia’; YMRS, ‘Young’s mania rating scale’; LM, ‘logical memory’; TMT, ‘trial making task’; DSB, ‘digital number substitution’; GAF, ‘global assessment of functioning’; P, ‘positive symptom’; N, ‘negative symptom’; G, ‘general psychopathology’; H, ‘Hamilton depression scale’.

Potential pathways from psychopathological symptoms to cognitive functions and finally to personal functioning were explored using directed acyclic graphing. In both discovery (Supplementary Table S7 and Figure 3a) and validation cohort (Supplementary Table S8 and Supplementary Figure S3), the syndrome-level network showed a pathway in which the disorganized factor cascade activated the excited, negative and positive factors, ultimately activating the YMRS. The disorganized factor also induced abnormal processing speed in the digital symbol substitution test. In contrast, logical memory and executive function exhibited a parallel relationship, with no intersection between them. These core depressive symptoms functioned as upstream drivers in directed acyclic graphs, sequentially activating downstream domains such as somatic complaints (e.g. fatigue, psychomotor changes), sleep disturbances (insomnia or hypersomnia), and comorbid anxiety symptoms.

The item-level causal relationship is quite consistent with the syndrome level. In item-level directed acyclic graph analysis, particularly within the discovery cohort (Supplementary Table S9 and Figure 3b), we found that negative symptoms cascade-activated cognitive function, while positive symptoms and depressive symptoms contributed to impairments in social functioning. In both the discovery and validation cohorts (Supplementary Table S10 and Supplementary Figure S4), item-level directed acyclic graph analyses, logical memory, and executive function exhibited a parallel relationship with no direct connection between them.

### Comparisons of networks between the sexes and disorders

In both the discovery and validation cohorts, comparison of networks at the syndrome level or the item level did not find any significant differences in invariance of network structure or global strength between males and females or between those with schizophrenia or bipolar disorder.

In the discovery cohort at the syndrome level (Supplementary Table S11), most pairwise connections (e.g. PosF-NegF, NegF-DisF) did not differ significantly between males and females, suggesting overall structural invariance between the sexes. Nevertheless, specific edges showed weak trends, such as DepF-Insomnia and Anxiety-Somatic, but these did not survive correction for multiple testing. Similarly, most pairwise connections did not differ significantly between schizophrenia and bipolar disorder (Supplementary Table S12); one exception was the DisF-Anxiety edge, while the connections PosF-ExcF and NegF-ExcF did not reach significance. Total connectivity did not differ systematically between the disorders, based on tests of global network strength invariance. Edges related to cognitive function, such as the edge linking immediate logical memory and part A of the trail-making test, varied subtly between disorders but not consistently across domains.

These results from the discovery cohort were echoed in the corresponding analyses of the validation cohort. In the gender-based network comparison, most pairwise connections (e.g. PosF-NegF, DisF-ExcF, YMRS-Depression) showed no significant differences between groups, indicating structural invariance across sexes (Supplementary Table S13). However, weak uncorrected trends emerged for specific edges: insomnia-TMT-B time, somatic-logical memory delayed, and negative symptoms-onset age. None survived multiple testing correction, suggesting no robust sex-specific pathway differences in the network. Similarly, most pairwise connections showed no significant differences between diagnostic groups (e.g. NegF-DisF, ExcF-DepF) (Supplementary Table S14). However, one edge demonstrated robust diagnostic specificity: PosF and YMRS connectivity differed significantly. Several symptom–symptom edges approached but did not reach significance after correction, including (DisF)-anxiety and ExcF-anxiety. Cognitive function edges showed limited diagnostic variation, with only insomnia-TMT-A time and disorganized features-DSB showing uncorrected differences. Global network strength invariance tests indicated no systemic connectivity differences between disorders.

## Discussion

Using a transdiagnostic network approach, the present study examined the complex relationships among psychotic symptoms, mood symptoms, cognitive dysfunction, and functional outcomes in schizophrenia and bipolar disorder. The two disorders, in men and women, appear to share a similar network architecture in which central roles are played by disorganized symptoms, as measured using the disorganized factor on the PANSS; negative symptoms, as measured using the negative factor on the PANSS; depressive symptoms, as measured using the depression factor on the HAMD; and cognitive deficits in processing speed. These findings align with the ‘dimensional’ perspective of severe mental disorders and support the idea that schizophrenia and bipolar disorder occupy overlapping positions along a psychopathological continuum instead of representing distinct categorical entities. This study advances our understanding of overlapping mechanisms in severe mental disorders, which may help personalize interventions.

### Symptoms at the core of schizophrenia and bipolar disorder

Disorganized and negative symptoms emerged as pivotal hubs in the psychopathological networks of schizophrenia and bipolar disorder, with strong connections to cognitive impairment (e.g. performance on the digital symbol substitution test) and functional disability as measured on the GAF and PSP. These findings are consistent with prior research showing that disorganization and negative symptoms had enduring correlation with functioning deterioration and persistent neurocognitive impairment (Olarewaju, Dumas, & Palaniyappan, 2023; Palaniyappan, 2022; Pelizza et al., 2021; Rathnaiah et al., 2020). Recent conceptualizations frame communication disorganization not merely as an individual cognitive deficit, but as a failure in interpersonal coordination – a disruption of the ‘social mind’ rooted in the bio-behavioral synchrony between interacting individuals (Olarewaju et al., 2023). Furthermore, disorganization and impoverishment together may reflect a core deficit in classical schizophrenia, associated with identifiable neurophysiological markers such as attenuated post-movement beta rebound, and contributing to persistent disability (Palaniyappan, 2022; Rathnaiah et al., 2020). Our results confirmed that disorganization and negative symptoms act as a bridge linking cognitive dysfunction to functional decline (Dey et al., 2021; Pan et al., 2021). Our observation that disorganized and negative factors on the PANSS were more central than traditional positive symptoms (e.g. hallucinations/delusions) in the disorder networks challenges the conventional ‘psychosis-centric model’ (Rodriguez-Jimenez et al., 2013). Therefore, therapeutic efforts should extend beyond the amelioration of positive symptoms to address these core pathophysiological processes, potentially through interventions that enhance interactive alignment, contextual processing, and inhibitory control.

We also found that depressed mood and loss of interest/pleasure (anhedonia) occupy central positions within the network of depressive symptoms, acting as pivotal hubs that influence other symptom clusters. In both the discovery and validation cohorts, the depression factors on the HAMD and PANSS emerged as upstream drivers in directed acyclic graphs, sequentially activating downstream domains such as somatic complaints (e.g. fatigue, psychomotor changes), sleep disturbances (insomnia or hypersomnia), and comorbid symptoms of anxiety. These findings imply the need to prioritize core depressive symptoms of low mood and anhedonia during interventions in order to prevent the emergence of somatic, anxiety, and sleep disturbances (Laumann et al., 2024).

### Disorganized symptoms drive deficits in processing speed and implications for treatment

Our network analysis revealed a robust association between processing speed deficits and disorganized symptoms, with directed acyclic graphing suggesting that disorganized symptoms drive abnormalities in processing speed. The negative correlation between cognitive performance and disorganized symptoms observed in our cohorts may reflect a shared pathology in the fronto-parietal circuit, as previous studies reported disorganization symptom (Das, Kumar, Francis, Liddle, & Palaniyappan, 2020; Li et al., 2019; Palaniyappan, Al-Radaideh, Gowland, & Liddle, 2020; Palaniyappan & Liddle, 2012) and processing speed (Hong et al., 2011) are respectively associated with frontal–parietal structure. Although previous studies have not explored the causal relationship between disorganization and cognitive deficits, our findings imply that interventions targeting conceptual disorganization or thought disorder may ameliorate deficits in processing speed (Minor et al., 2015). In this way, our findings suggest an answer to the previously posed question of whether cognitive improvement can be achieved merely by mitigating clinical symptoms or whether interventions should target the cognitive deficits directly (Chavez-Baldini et al., 2023). Indeed, our failure to detect associations of symptom severity with performance on the trail-making test or logical memory test implies the usefulness of domain-specific cognitive remediation strategies (e.g. training in attention or executive function) in parallel with symptom-focused interventions (Trapp et al., 2022). Our findings also highlight the modular organization of cognitive systems: logical memory and executive functions operate through distinct, non-interacting pathways. This parallel architecture suggests that these domains may require separate intervention frameworks rather than integrated approaches. For instance, memory rehabilitation programs targeting hippocampal networks (Squire & Wixted, 2011) and executive training focusing on prefrontal circuits (Cristofori, Cohen-Zimerman, & Grafman, 2019) may complement each other well.

## Functional impairment: converging pathways

Our network analyses at the syndrome and item levels revealed nuanced relationships among psychopathological symptoms, cognitive profiles, and functional outcomes. In the discovery cohort, syndrome-level analysis found that score on the GAF was associated negatively with negative symptoms on the PANSS but positively with performance on the digital symbol substitution test. At the same time, directed acyclic graphing did not detect causal links between psychopathology or processing speed on one hand and GAF score on the other. While a lower GAF score may reflect depression and psychosis severity, the disorganized symptoms, negative symptoms, as well as depressive symptoms captured in such functional assessments are themselves shaped by social functioning (Hinzen & Palaniyappan, 2024; McKnight & Kashdan, 2009; Palaniyappan, 2021).

Nevertheless, directed acyclic graphing at the item level suggests that delusions (item P1 on the PANSS) and impaired work/activities (item 7 on the HAMD) may drive deterioration on the GAF. These findings align with the ‘symptom-specific functional disruption’ hypothesis, according to which certain core symptoms (e.g. reality distortion, motivational deficits) may act through distinct neural pathways (e.g. delusions may leave patients unsure on how to behave in social interactions, while depression may reduce participation in everyday activities), to exert disproportionately strong effects on psychosocial functioning (Christie, Inman, Davys, & Cook, 2021; Heering & van Haren, 2016; McKnight & Kashdan, 2009).

The validation cohort findings further refined this symptom–network model. Network analysis at the syndrome level positioned disorganized symptoms as a central hub that activated positive and negative clusters of symptoms that, together, impaired score on PSP. Directed acyclic graphing revealed the following cascading pathway: disorganized symptoms → excited factor (e.g. agitation) → Young’s mania symptoms→ positive/negative symptoms → PSP decline. This hierarchical structure implies that targeting disorganized symptoms may disrupt downstream cascades of pathological symptoms (Barlati et al., 2013).

Though we found the digital symbol substitution test and trail making test measures were associated with functional outcomes in network analyses, yet were absent from directed acyclic graphs, raises the possibility that cognitive deficits may be disease manifestations concomitant and parallel with functioning deficits, rather than direct mediators between symptoms and functioning. This should be explored further because it has critical implications for designing interventions (Kalisova et al., 2023). Such work could help identify predictors of poor functioning in schizophrenia and bipolar disorders, which may improve diagnosis and management.

## Stability of symptom networks across medication status and sex differences

Regarding medication status, while the potential confounding effect of pharmacotherapy in bipolar disorder patients cannot be entirely ruled out, the striking similarity in network architecture between medication-naïve schizophrenia patients and medicated bipolar disorder patients – as well as between first-episode and chronic schizophrenia subgroups – suggests that the identified core symptom relationships (e.g. disorganization, depression) may represent stable, transdiagnostic features less susceptible to medication effects. As for the observed sex differences, the superior performance of females on the digital symbol substitution test is consistent with prior literature suggesting slight neurocognitive advantages in processing speed tasks (Siedlecki, Falzarano, & Salthouse, 2019). While the later illness onset in males might be due to the cross-sectional nature of our study design and the results might be influenced by cohort effects. These demographic and clinical variations, however, did not significantly influence the overall network structure or centrality estimates, supporting the robustness of our transdiagnostic findings.

### Limitations and future directions

Our cross-sectional study inferred causal associations from directed acyclic graphing, while the symptom of disorganization can change over time, so its findings should be validated and extended in longitudinal studies (Alonso-Sánchez et al., 2022). We cannot exclude confounding of our analyses by ongoing medication: first-episode schizophrenia patients in our study were medication-naïve, but those with chronic schizophrenia or bipolar disorder were on pharmacotherapy. Nevertheless, such confounding may not have impacted our analysis much, given that network analysis showed similar results between individuals with first-episode or chronic schizophrenia. Future investigations should prioritize studying medication-naïve BD patients or those in a medication washout period.

Disorganization is best measured using language samples because speech directly reflects thought processes, and computational natural language processing (NLP) can objectively quantify subtle, clinically significant deviations that traditional rating scales may miss (Alonso-Sánchez, Limongi, Gati, & Palaniyappan, 2023; Dalal et al., 2025). Future work should focus on longitudinal, cross-linguistic studies, integrating NLP markers with neurobiological data (Mehta et al., 2021), and developing personalized intervention (Corona Hernández et al., 2023).

## Conclusion

By mapping the intricate associations among symptoms, cognition, and functioning, this study advances the dimensional conceptualization of severe mental disorders. The similarity in network architecture between schizophrenia and bipolar disorder challenges the traditional categorical approach to diagnosing and treating the disorders. Our results identify disorganization, depressive symptoms, and processing speed as therapeutic priorities when personalizing interventions. They advocate for a paradigm shift toward transdiagnostic, network-informed treatments aimed at disrupting core pathways in severe mental disorders.

## Supporting information

Yu et al. supplementary material Yu et al. supplementary material

## Supplementary material

The supplementary material for this article can be found at http://doi.org/10.1017/S0033291725102481.

## References

[r1] Alonso-Sánchez, M. F., Ford, S. D., MacKinley, M., Silva, A., Limongi, R., & Palaniyappan, L. (2022). Progressive changes in descriptive discourse in first episode schizophrenia: A longitudinal computational semantics study. Schizophrenia (Heidelberg), 8(1), 36. 10.1038/s41537-022-00246-8. PMC9261094 35853894

[r2] Alonso-Sánchez, M. F., Limongi, R., Gati, J., & Palaniyappan, L. (2023). Language network self-inhibition and semantic similarity in first-episode schizophrenia: A computational-linguistic and effective connectivity approach. Schizophrenia Research, 259, 97–103. 10.1016/j.schres.2022.04.007. 35568676

[r3] Barlati, S., Deste, G., De Peri, L., Ariu, C., & Vita, A. (2013). Cognitive remediation in schizophrenia: Current status and future perspectives. Schizophrenia Research and Treatment, 2013, 156084. 10.1155/2013/156084. 24455253 PMC3877646

[r4] Batool, K., & Niazi, M. A. (2014). Towards a methodology for validation of centrality measures in complex networks. PLoS One, 9(4), e90283. 10.1371/journal.pone.0090283. 24709999 PMC3977855

[r5] Bonnín, C. D. M., Reinares, M., Martínez-Arán, A., Jiménez, E., Sánchez-Moreno, J., Solé, B., … Vieta, E. (2019). Improving functioning, quality of life, and well-being in patients with bipolar disorder. The International Journal of Neuropsychopharmacology, 22(8), 467–477. 10.1093/ijnp/pyz018. 31093646 PMC6672628

[r6] Chavez-Baldini, U., Nieman, D. H., Keestra, A., Lok, A., Mocking, R. J. T., de Koning, P., & Denys, D. (2023). The relationship between cognitive functioning and psychopathology in patients with psychiatric disorders: A transdiagnostic network analysis. Psychological Medicine, 53(2), 476–485. 10.1017/S0033291721001781. 34165065 PMC9899564

[r7] Christie, L., Inman, J., Davys, D., & Cook, P. A. (2021). A systematic review into the effectiveness of occupational therapy for improving function and participation in activities of everyday life in adults with a diagnosis of depression. Journal of Affective Disorders, 282, 962–973. 10.1016/j.jad.2020.12.080. 33601741

[r8] Corona Hernández, H., Corcoran, C., Achim, A. M., de Boer, J. N., Boerma, T., Brederoo, S. G.,… Palaniyappan, L. (2023). Natural language processing markers for psychosis and other psychiatric disorders: Emerging themes and research agenda From a cross-linguistic workshop. Schizophrenia Bulletin, 49(Suppl_2), S86–s92. 10.1093/schbul/sbac215 36946526 PMC10031727

[r9] Cristofori, I., Cohen-Zimerman, S., & Grafman, J. (2019). Executive functions. Handbook of Clinical Neurology, 163, 197–219. 10.1016/b978-0-12-804281-6.00011-2. 31590731

[r10] Dalal, T. C., Liang, L., Silva, A. M., Mackinley, M., Voppel, A., & Palaniyappan, L. (2025). Speech based natural language profile before, during and after the onset of psychosis: A cluster analysis. Acta Psychiatrica Scandinavica, 151(3), 332–347. 10.1111/acps.13685. 38600593 PMC11787926

[r11] Das, T. K., Kumar, J., Francis, S., Liddle, P. F., & Palaniyappan, L. (2020). Parietal lobe and disorganisation syndrome in schizophrenia and psychotic bipolar disorder: A bimodal connectivity study. Psychiatry Research: Neuroimaging, 303, 111139. 10.1016/j.pscychresns.2020.111139. 32707490

[r12] Dey, A., Dempster, K., MacKinley, M., Jeon, P., Das, T., Khan, A., & Palaniyappan, L. (2021). Conceptual disorganization and redistribution of resting-state cortical hubs in untreated first-episode psychosis: A 7T study. NPJ Schizophrenia, 7(1), 4. 10.1038/s41537-020-00130-3. 33500416 PMC7838254

[r13] Epskamp, S., Borsboom, D., & Fried, E. I. (2018). Estimating psychological networks and their accuracy: A tutorial paper. Behavior Research Methods, 50(1), 195–212. 10.3758/s13428-017-0862-1. 28342071 PMC5809547

[r14] Fett, A.-K. J., Velthorst, E., Reichenberg, A., Ruggero, C. J., Callahan, J. L., Fochtmann, L. J., & Kotov, R. (2020). Long-term changes in cognitive functioning in individuals with psychotic disorders: Findings From the Suffolk County mental health project. JAMA Psychiatry, 77(4), 387–396. 10.1001/jamapsychiatry.2019.3993. 31825511 PMC6990826

[r15] Heering, H. D., & van Haren, N. E. (2016). Social functioning in patients with a psychotic disorder and first rank symptoms. Psychiatry Research, 237, 147–152. 10.1016/j.psychres.2016.01.050. 26892072

[r16] Hinzen, W., & Palaniyappan, L. (2024). The ‘L-factor’: Language as a transdiagnostic dimension in psychopathology. Progress in Neuro-Psychopharmacology & Biological Psychiatry, 131, 110952. 10.1016/j.pnpbp.2024.110952. 38280712

[r17] Hong, L. E., Schroeder, M., Ross, T. J., Buchholz, B., Salmeron, B. J., Wonodi, I., & Stein, E. A. (2011). Nicotine enhances but does not normalize visual sustained attention and the associated brain network in schizophrenia. Schizophrenia Bulletin, 37(2), 416–425. 10.1093/schbul/sbp089. 19713300 PMC3044635

[r18] Huang, Y. H., Liu, C., Zhang, J. B., Li, S. B., Wang, L. L., Hu, H. X.,. Chan, R. C. K. (2024). A transdiagnostic network analysis of childhood trauma and psychopathology. Schizophrenia Bulletin. 10.1093/schbul/sbae137 PMC12061646 39148412

[r19] Izquierdo, A., Cabello, M., Leal, I., Mellor-Marsá, B., Ayora, M., Bravo-Ortiz, M.-F., & Albarracin-García, L. (2021). The interplay between functioning problems and symptoms in first episode of psychosis: An approach from network analysis. Journal of Psychiatric Research, 136, 265–273. 10.1016/j.jpsychires.2021.02.024. 33621912

[r20] Jiménez, S., de Montis, I., & Garza-Villarreal, E. A. (2025). Longitudinal dynamics between the central nodes in the symptoms network of borderline personality disorder: An intraindividual network analysis. Journal of Affective Disorders, 372, 431–439. 10.1016/j.jad.2024.12.005. 39672474

[r21] Kalisova, L., Michalec, J., Dechterenko, F., Silhan, P., Hyza, M., Chlebovcova, M., & Bezdicek, O. (2023). Impact of cognitive performance and negative symptoms on psychosocial functioning in Czech schizophrenia patients. Schizophrenia, 9(1), 43. 10.1038/s41537-023-00374-9. 37460587 PMC10352309

[r22] Laumann, L. E., Lee, J., Blackmon, J. E., Delcourt, M. L., Sullivan, M. C., Cruess, S. E., & Cruess, D. G. (2024). Depression and anxiety as mediators of the relationship between sleep disturbance and somatic symptoms among adolescents on a psychiatric inpatient unit. Clinical Child Psychology and Psychiatry, 29(2), 513–525. 10.1177/13591045231198365. 37669806

[r23] Li, M., Li, X., Das, T. K., Deng, W., Li, Y., Zhao, L., & Li, T. (2019). Prognostic utility of multivariate Morphometry in schizophrenia. Frontiers in Psychiatry, 10, 245. 10.3389/fpsyt.2019.00245. 31037060 PMC6476259

[r24] Limongi, R., Silva, A. M., Mackinley, M., Ford, S. D., & Palaniyappan, L. (2023). Active inference, epistemic value, and uncertainty in conceptual disorganization in first-episode schizophrenia. Schizophrenia Bulletin, 49(Suppl_2), S115–s124. 10.1093/schbul/sbac125 36946528 PMC10031740

[r25] Lipsky, A. M., & Greenland, S. (2022). Causal directed acyclic graphs. JAMA, 327(11), 1083–1084. 10.1001/jama.2022.1816. 35226050

[r26] Mackinley, M., Limongi, R., Silva, A. M., Richard, J., Subramanian, P., Ganjavi, H., & Palaniyappan, L. (2023). More than words: Speech production in first-episode psychosis predicts later social and vocational functioning. Frontiers in Psychiatry, 14, 1144281. 10.3389/fpsyt.2023.1144281. 37124249 PMC10140590

[r27] MacQueen, G. M., & Memedovich, K. A. (2017). Cognitive dysfunction in major depression and bipolar disorder: Assessment and treatment options. Psychiatry and Clinical Neurosciences, 71(1), 18–27. 10.1111/pcn.12463. 27685435

[r28] Martinez-Aran, A., & Vieta, E. (2015). Cognition as a target in schizophrenia, bipolar disorder and depression. European Neuropsychopharmacology, 25(2), 151–157. 10.1016/j.euroneuro.2015.01.007. 25661911

[r29] Martinez-Aran, A., Vieta, E., Torrent, C., Sanchez-Moreno, J., Goikolea, J. M., Salamero, M., & Ayuso-Mateos, J. L. (2007). Functional outcome in bipolar disorder: The role of clinical and cognitive factors. Bipolar Disorders, 9(1–2), 103–113. 10.1111/j.1399-5618.2007.00327.x. 17391354

[r30] McKnight, P. E., & Kashdan, T. B. (2009). The importance of functional impairment to mental health outcomes: A case for reassessing our goals in depression treatment research. Clinical Psychology Review, 29(3), 243–259. 10.1016/j.cpr.2009.01.005. 19269076 PMC2814224

[r31] McNally, R. J., Heeren, A., & Robinaugh, D. J. (2017). A Bayesian network analysis of posttraumatic stress disorder symptoms in adults reporting childhood sexual abuse. European Journal of Psychotraumatology, 8(Suppl. 3), 1341276. 10.1080/20008198.2017.1341276. 29038690 PMC5632780

[r32] Mehta, U. M., Punith, M., Kumar, C. N., Kumar, J. K., Reddy, Y. J., & Thirthalli, J. (2021). Dissimilar social cognition signatures in remitted schizophrenia and bipolar disorder. Asian Journal of Psychiatry, 57, 102593. 10.1016/j.ajp.2021.102593. 33581371

[r33] Merikangas, K. R., Jin, R., He, J. P., Kessler, R. C., Lee, S., Sampson, N. A., & Zarkov, Z. (2011). Prevalence and correlates of bipolar spectrum disorder in the world mental health survey initiative. Archives of General Psychiatry, 68(3), 241–251. 10.1001/archgenpsychiatry.2011.12. 21383262 PMC3486639

[r34] Minor, K. S., Marggraf, M. P., Davis, B. J., Luther, L., Vohs, J. L., Buck, K. D., & Lysaker, P. H. (2015). Conceptual disorganization weakens links in cognitive pathways: Disentangling neurocognition, social cognition, and metacognition in schizophrenia. Schizophrenia Research, 169(1), 153–158. 10.1016/j.schres.2015.09.026. 26441007

[r35] Monrad Aas, I H. (2014). Collecting information for rating global assessment of functioning (GAF): Sources of information and methods for information collection. Current Psychiatry Reviews, 10(4), 330–347. 10.2174/1573400509666140102000243. 25598769 PMC4287015

[r36] Moroń, M., Mengel-From, J., Zhang, D., Hjelmborg, J., & Semkovska, M. (2025). Depressive symptoms, cognitive functions and daily activities: An extended network analysis in monozygotic and dizygotic twins. Journal of Affective Disorders, 368, 398–409. 10.1016/j.jad.2024.09.089. 39299594

[r37] Morosini, P. L., Magliano, L., Brambilla, L., Ugolini, S., & Pioli, R. (2000). Development, reliability and acceptability of a new version of the DSM-IV social and occupational functioning assessment scale (SOFAS) to assess routine social functioning. Acta Psychiatrica Scandinavica, 101(4), 323–329. 10782554

[r38] Olarewaju, E., Dumas, G., & Palaniyappan, L. (2023). Disorganized communication and social dysfunction in schizophrenia: Emerging concepts and methods. Current Psychiatry Reports, 25(11), 671–681. 10.1007/s11920-023-01462-4. 37740852

[r39] Palaniyappan, L. (2021). More than a biomarker: Could language be a biosocial marker of psychosis? NPJ Schizophrenia, 7(1), 42. 10.1038/s41537-021-00172-1. 34465778 PMC8408150

[r40] Palaniyappan, L. (2022). Dissecting the neurobiology of linguistic disorganisation and impoverishment in schizophrenia. Seminars in Cell & Developmental Biology, 129, 47–60. 10.1016/j.semcdb.2021.08.015. 34507903

[r41] Palaniyappan, L., Al-Radaideh, A., Gowland, P. A., & Liddle, P. F. (2020). Cortical thickness and formal thought disorder in schizophrenia: An ultra high-field network-based morphometry study. Progress in Neuro-Psychopharmacology & Biological Psychiatry, 101, 109911. 10.1016/j.pnpbp.2020.109911. 32151693

[r42] Palaniyappan, L., & Liddle, P. F. (2012). Dissociable morphometric differences of the inferior parietal lobule in schizophrenia. European Archives of Psychiatry and Clinical Neuroscience, 262(7), 579–587. 10.1007/s00406-012-0314-y. 22454243

[r43] Pan, Y., Dempster, K., Jeon, P., Théberge, J., Khan, A. R., & Palaniyappan, L. (2021). Acute conceptual disorganization in untreated first-episode psychosis: A combined magnetic resonance spectroscopy and diffusion imaging study of the cingulum. Journal of Psychiatry & Neuroscience, 46(3), E337–e346. 10.1503/jpn.200167. 33904669 PMC8327974

[r44] Pelizza, L., Leuci, E., Maestri, D., Quattrone, E., Azzali, S., Paulillo, G., & Raballo, A. (2021). Disorganization in first episode schizophrenia: Treatment response and psychopathological findings from the 2-year follow-up of the “Parma early psychosis, program”. Journal of Psychiatric Research, 141, 293–300. 10.1016/j.jpsychires.2021.07.015. 34274840

[r45] Perlis, R. H., Brown, E., Baker, R. W., & Nierenberg, A. A. (2006). Clinical features of bipolar depression versus major depressive disorder in large multicenter trials. The American Journal of Psychiatry, 163(2), 225–231. 10.1176/appi.ajp.163.2.225. 16449475

[r46] Picco, L., Lau, Y. W., Pang, S., Jeyagurunathan, A., Vaingankar, J. A., Abdin, E., & Subramaniam, M. (2018). Predictors of general functioning and correlates of quality of life: A cross-sectional study among psychiatric outpatients. Annals of the Academy of Medicine, Singapore, 47(1), 3–12. 29493706

[r47] Qiao, Z., Lafit, G., Lecei, A., Achterhof, R., Kirtley, O. J., Hiekkaranta, A. P., & van Winkel, R. (2024). Childhood adversity and emerging psychotic experiences: A network perspective. Schizophrenia Bulletin, 50(1), 47–58. 10.1093/schbul/sbad079. 37318106 PMC10754171

[r48] Rathnaiah, M., Liddle, E. B., Gascoyne, L., Kumar, J., Zia Ul Haq Katshu, M., Faruqi, C., & Liddle, P. F. (2020). Quantifying the Core deficit in classical schizophrenia. Schizophrenia Bulletin Open, 1(1), sgaa031. 10.1093/schizbullopen/sgaa031. 32803162 PMC7418866

[r49] Robinaugh, D. J., Millner, A. J., & McNally, R. J. (2016). Identifying highly influential nodes in the complicated grief network. Journal of Abnormal Psychology, 125(6), 747–757. 10.1037/abn0000181. 27505622 PMC5060093

[r50] Rodriguez-Jimenez, R., Bagney, A., Mezquita, L., Martinez-Gras, I., Sanchez-Morla, E. M., Mesa, N., & Palomo, T. (2013). Cognition and the five-factor model of the positive and negative syndrome scale in schizophrenia. Schizophrenia Research, 143(1), 77–83. 10.1016/j.schres.2012.10.020. 23201306

[r51] Saha, S., Chant, D., & McGrath, J. (2007). A systematic review of mortality in schizophrenia: Is the differential mortality gap worsening over time? Archives of General Psychiatry, 64(10), 1123–1131. 10.1001/archpsyc.64.10.1123. 17909124

[r52] Saleh, A., Potter, G. G., McQuoid, D. R., Boyd, B., Turner, R., MacFall, J. R., & Taylor, W. D. (2017). Effects of early life stress on depression, cognitive performance and brain morphology. Psychological Medicine, 47(1), 171–181. 10.1017/s0033291716002403. 27682320 PMC5195852

[r53] Shafer, A. B. (2006). Meta-analysis of the factor structures of four depression questionnaires: Beck, CES-D, Hamilton, and Zung. Journal of Clinical Psychology, 62(1), 123–146. 10.1002/jclp.20213. 16287149

[r54] Shao, W., Simmonds-Buckley, M., Zavlis, O., & Bentall, R. P. (2024). The common structure of the major psychoses: More similarities than differences in the network structures of schizophrenia, schizoaffective disorder, and psychotic bipolar disorder. Schizophrenia Bulletin. 10.1093/schbul/sbae154. PMC12236324 39259601

[r55] Sheffield, J. M., Karcher, N. R., & Barch, D. M. (2018). Cognitive deficits in psychotic disorders: A lifespan perspective. Neuropsychology Review, 28(4), 509–533. 10.1007/s11065-018-9388-2. 30343458 PMC6475621

[r56] Siedlecki, K. L., Falzarano, F., & Salthouse, T. A. (2019). Examining gender differences in neurocognitive functioning across adulthood. Journal of the International Neuropsychological Society, 25(10), 1051–1060. 10.1017/s1355617719000821. 31378214 PMC7331091

[r57] Silva, A. M., Limongi, R., MacKinley, M., Ford, S. D., Alonso-Sánchez, M. F., & Palaniyappan, L. (2023). Syntactic complexity of spoken language in the diagnosis of schizophrenia: A probabilistic Bayes network model. Schizophrenia Research, 259, 88–96. 10.1016/j.schres.2022.06.011. 35752547

[r58] Squire, L. R., & Wixted, J. T. (2011). The cognitive neuroscience of human memory since H.M. Annual Review of Neuroscience, 34, 259–288. 10.1146/annurev-neuro-061010-113720. PMC3192650 21456960

[r59] Sun, H.-L., He, F., Rao, W.-W., Qi, Y., Rao, S.-Y., Ho, T. I., & Xiang, Y.-T. (2025). Gender differences in behavioral and emotional problems among school children and adolescents in China: National survey findings from a comparative network perspective. Journal of Affective Disorders, 369, 227–233. 10.1016/j.jad.2024.09.067. 39284529

[r60] Tianmei, S., Liang, S., Yun’ai, S., Chenghua, T., Jun, Y., Jia, C., & Hongyan, Z. (2011). The Chinese version of the personal and social performance scale (PSP): Validity and reliability. Psychiatry Research, 185(1–2), 275–279. 10.1016/j.psychres.2010.05.001. 20542575

[r61] Trapp, W., Heid, A., Röder, S., Wimmer, F., & Hajak, G. (2022). Cognitive remediation in psychiatric disorders: State of the evidence, future perspectives, and some bold ideas. Brain Sciences, 12(6). 10.3390/brainsci12060683. PMC9221116 35741569

[r62] Tsouvalas, T., Konstantinidou, L., Georgiou, G., Birmpili, E. M., & Nikolara, R. (2011). Personal and social performance scale (PSP) in community-based patients with serious mental illness. European Psychiatry, 26(S2), 1517–1517. 10.1016/S0924-9338(11)73221-7.

[r63] Upthegrove, R., Marwaha, S., & Birchwood, M. (2017). Depression and schizophrenia: Cause, consequence, or trans-diagnostic issue? Schizophrenia Bulletin, 43(2), 240–244. 10.1093/schbul/sbw097. 27421793 PMC5605248

[r64] van Borkulo, C. D., van Bork, R., Boschloo, L., Kossakowski, J. J., Tio, P., Schoevers, R. A., & Waldorp, L. J. (2023). Comparing network structures on three aspects: A permutation test. Psychological Methods, 28(6), 1273–1285. 10.1037/met0000476. 35404628

[r65] Wallwork, R. S., Fortgang, R., Hashimoto, R., Weinberger, D. R., & Dickinson, D. (2012). Searching for a consensus five-factor model of the positive and negative syndrome scale for schizophrenia. Schizophrenia Research, 137(1–3), 246–250. 10.1016/j.schres.2012.01.031. 22356801 PMC3351536

[r66] Young, R. C., Biggs, J. T., Ziegler, V. E., & Meyer, D. A. (1978). A rating scale for mania: Reliability, validity and sensitivity. The British Journal of Psychiatry, 133, 429–435. 10.1192/bjp.133.5.429. 728692

[r67] Zhang, Z. (2016). Multiple imputation with multivariate imputation by chained equation (MICE) package. Annals of Translational Medicine, 4(2), 30. 10.3978/j.issn.2305-5839.2015.12.63. 26889483 PMC4731595

