# Supplementary material for: Network analysis of relationships among psychopathology, cognitive function, and psychosocial functioning in independent samples of Chinese with schizophrenia or bipolar disorder
Source: Psychol Med. 2025 Nov 20;55:e353. doi: 10.1017/S0033291725102481 (PMC13058621; doi:10.1017/S0033291725102481)
Supplement: Yu et al. supplementary material [file S0033291725102481sup001.docx]

**Supplementary Materials**

**Supplementary Results**

**Demographic and clinical variables of the Validation cohort**

Supplementary Table 1 summarizes demographic, clinical, and cognitive characteristics stratified by sex in a psychiatric cohort (N=235, 164 males, 71 females). Males were significantly older (28.25 vs. 24.28 years, p<0.001) with later illness onset (22.37 vs. 19.15 years, p=0.002). Females exhibited higher severity in PANSS negative (p=0.033) and depressed (p<0.001) factors, as well as elevated HAMD anxiety (p=0.042) and depression (p=0.004) scores. Males scored higher on YMRS (mania severity, p=0.007). No significant sex differences were observed in education, PANSS positive/disorganized/excited factors, cognitive performance (TMT-A/B, DSB, logical memory), or psychosocial functioning (PSP).

Supplemenatry Table 2 compares demographic, clinical, and cognitive profiles between schizophrenia (SCZ, n=138) and bipolar disorder (BD, n=97) patients. SCZ patients exhibited significantly higher PANSS positive (p<0.001), negative (p<0.001), and disorganized (p<0.001) factor scores, reflecting core psychotic symptoms, while BD patients showed elevated YMRS (mania severity, p<0.001) and HAMD anxiety (p=0.028)/insomnia (p=0.008) scores. Cognitive performance differed marginally, with BD patients outperforming SCZ in DSB (p=0.004) and PSP (p=0.021). No group differences were observed in age, education, illness duration, age of onset, TMT-A/B, or logical memory. Diagnoses showed balanced sex distribution.

**Supplementary Tables**

**Supplementary Table 1 Descriptive Summary of the Participants and Gender Group Effects of the Validation cohort**

|  | **Total** | **Male (164)** | **Female (71)** | **χ^2^/t** | **P** | **φc/Cohen’s**  ***d*** |
| --- | --- | --- | --- | --- | --- | --- |
| Age | 27.05 (8.40) | 28.25 (8.37) | 24.28 (7.84) | 3.40 | **<0.001** | 8.22 |
| Education Years | 13.11 (7.49) | 13.46 (8.65) | 12.25 (3.26) | 1.09 | 0.28 | 7.49 |
| Duration of illness | 62.94 (77.07) | 67.04 (77.00) | 53.21 (77.06) | 1.21 | 0.20 | 77.00 |
| Age of onset (years) | 21.42 (7.00) | 22.37 (7.34) | 19.15 (5.36) | 3.19 | **0.002** | 6.93 |
| **Diagnosis** |  |  |  | 0.44 | 0.51 | 0.043 |
| SCZ | 138 | 94 | 44 |  |  |  |
| BD | 97 | 70 | 27 |  |  |  |
| **PANSS** |  |  |  |  |  |  |
| Positive factor | 10.43 (4.87) | 10.76 (4.80) | 9.68 (4.98) | 1.57 | 0.12 | 4.86 |
| Negative factor | 11.88 (6.51) | 11.28 (6.13) | 13.25 (7.18) | -2.15 | **0.033** | 4.98 |
| Disorganized factor | 6.06 (2.64) | 6.10 (2.57) | 5.94 (2.80) | 0.43 | 0.67 | 2.64 |
| Excited factor | 7.63 (3.79) | 7.89 (3.68) | 7.04 (4.01) | 1.58 | 0.12 | 3.78 |
| Depressed factor | 5.91 (2.81) | 5.51 (2.56) | 6.85 (3.16) | -3.43 | **<0.001** | 2.75 |
| **HAMD** |  |  |  |  |  |  |
| Anxiety | 2.80 (2.26) | 2.60 (2.13) | 3.25 (2.49) | -2.04 | 0.042 | 2.24 |
| Depression | 3.34 (3.52) | 2.90 (3.24) | 4.35 (3.91) | -2.95 | **0.004** | 3.46 |
| Insomnia | 1.07 (1.58) | 0.95 (1.49) | 1.34 (1.75) | -1.74 | 0.084 | 1.57 |
| Somatic symptoms | 0.67 (1.15) | 0.59 (1.04) | 0.86 (1.37) | -1.65 | 0.099 | 1.15 |
| **YMRS** | 11.52 (9.40) | 12.63 (9.68) | 8.71 (8.06) | 2.74 | **0.007** | 9.26 |
| **Cognitive function** |  |  |  |  |  |  |
| TMT-A time | 44.55 (23.33) | 44.77 (19.75) | 44.02 (30.60) | 0.21 | 0.84 | 23.38 |
| TMT-B time | 73.12 (36.36) | 75.55 (39.10) | 67.15 (27.91) | 1.49 | 0.14 | 36.25 |
| DSB | 47.92 (15.06) | 47.14 (14.95) | 49.83 (15.28) | -1.16 | 0.25 | 15.05 |
| Logical memory immediately | 7.02 (4.18) | 6.92 (4.22) | 7.29 (4.10) | -0.57 | 0.57 | 4.19 |
| Logical memory delayed | 4.75 (3.48) | 4.64 (3.44) | 5.02 (3.60) | -0.70 | 0.49 | 3.49 |
| **PSP** | 58.87 (19.14) | 59.45 (18.76) | 57.51 (20.06) | 0.71 | 0.48 | 19.16 |

Note: P < .05 are bold. SCZ,schizophrenia; BD, bipolar disorder; PANSS, the Positive and Negative Symptom Scale; HAMD, Hamilton depression scale; YMRS, Young mania rating scale; TMT, trail making test; DSB, digital symbol substitution; PSP, personal and social performance.

**Supplementary Table 2 Comparison of Demographic and Clinical Information Among the Diagnostic Groups of the Validation cohort**

|  | **Total** | **SCZ (138)** | **BD (97)** | ***K*^2^/t** | **P** | **φc/Cohen’s**  ***d*** |
| --- | --- | --- | --- | --- | --- | --- |
| Age | 27.05 (8.40) | 26.42 (7.76) | 27.95 (9.21) | -1.38 | 0.17 | 8.16 |
| Education Years | 13.11 (7.49) | 13.17 (9.44) | 13.02 (2.91) | 0.15 | 0.88 | 7.51 |
| Duration of illness (months) | 62.94 (77.07) | 57.83 (77.36) | 70.38 (76.47) | -1.18 | 0.24 | 77.00 |
| Age of onset (years) | 21.42 (7.00) | 21.19 (6.14) | 21.74 (8.03) | -0.57 | 0.57 | 6.97 |
| **Diagnosis** |  |  |  | 0.44 | 0.51 | 0.043 |
| Male | 164 | 94 | 70 |  |  |  |
| Female | 71 | 44 | 27 |  |  |  |
| **PANSS** |  |  |  |  |  |  |
| Positive factor | 10.43 (4.87) | 12.36 (4.39) | 7.69 (4.17) | 8.18 | **<0.001** | 4.30 |
| Negative factor | 11.88 (6.51) | 14.14 (6.98) | 8.66 (4.01) | 6.96 | **<0.001** | 5.94 |
| Disorganized factor | 6.06 (2.64) | 6.67 (2.70) | 5.19 (2.28) | 4.41 | **<0.001** | 2.54 |
| Excited factor | 7.63 (3.79) | 7.75 (3.97) | 7.47 (3.53) | 0.54 | 0.59 | 3.80 |
| Depressed factor | 5.91 (2.81) | 5.91 (2.69) | 5.92 (3.00) | -0.31 | 0.98 | 2.82 |
| **HAMD** |  |  |  |  |  |  |
| Anxiety | 2.80 (2.26) | 2.53 (2.20) | 3.19 (2.31) | -2.21 | **0.028** | 2.24 |
| Depression | 3.34 (3.52) | 3.09 (2.94) | 3.71 (4.20) | -1.33 | 0.18 | 3.51 |
| Insomnia | 1.07 (1.58) | 0.84 (1.48) | 1.40 (1.66) | -2.69 | **0.008** | 1.56 |
| Somatic symptoms | 0.67 (1.15) | 0.55 (0.96) | 0.84 (1.36) | -1.92 | 0.056 | 1.15 |
| **YMRS** | 11.52 (9.40) | 7.97 (6.08) | 16.07 (10.86) | -6.76 | **<0.001** | 8.52 |
| **Cognitive function** |  |  |  |  |  |  |
| TMT-A time | 44.55 (23.33) | 46.55 (24.95) | 41.89 (20.80) | 1.40 | 0.16 | 23.27 |
| TMT-B time | 73.12 (36.36) | 74.12 (37.39) | 71.79 (35.10) | 0.45 | 0.66 | 36.43 |
| DSB | 47.92 (15.06) | 45.32 (14.39) | 51.40 (15.32) | -2.90 | **0.004** | 14.79 |
| Logical memory immediately | 7.02 (4.18) | 6.74(4.03) | 7.41 (4.37) | -1.15 | 0.25 | 4.18 |
| Logical memory delayed | 4.75 (3.48) | 4.72 (3.59) | 4.79 (3.36) | -0.14 | 0.89 | 3.49 |
| **PSP** | 58.87 (19.14) | 56.43 (17.74) | 62.29 (20.55) | -2.32 | **0.021** | 18.96 |

Note: P < .05 are bold. SCZ,schizophrenia; BD, bipolar disorder; PANSS, the Positive and Negative Syndrome Scale; HAMD, Hamilton depression scale; YMRS, Young mania rating scale; TMT, trail making test; DSB, digital symbol substitution; PSP, personal and social performance.

**Supplementary Table 3** **Centrality, predictability, and expected influence of nodes in the dimension-level network of the transdiagnostic sample of the discovery cohort.**

|  | **Strength** | **Closeness** | **Betweenness** | **Expected Influence** | **Predictability** |
| --- | --- | --- | --- | --- | --- |
| **PANSS** |  |  |  |  |  |
| Positive factor | 1.009461 | 0.004422 | 4 | 0.554854 | 0.681 |
| Negative factor | 1.005798 | 0.004995 | 32 | 0.45199 | 0.667 |
| Disorganized factor | 1.185468 | 0.005352 | 72 | 0.527081 | 0.608 |
| Excited factor | 1.111399 | 0.004851 | 28 | 0.674453 | 0.648 |
| Depressed factor | 0.970555 | 0.004473 | 30 | 0.852953 | 0.645 |
| **HAMD** |  |  |  |  |  |
| Anxiety | 0.85559 | 0.004191 | 12 | 0.85559 | 0.773 |
| Depression | 1.30635 | 0.00461 | 46 | 0.696027 | 0.591 |
| Insomnia | 0.561741 | 0.003284 | 0 | 0.561741 | 0.834 |
| Somatic | 0.894605 | 0.004096 | 24 | 0.784532 | 0.742 |
| **YMRS** | 0.804912 | 0.004521 | 18 | 0.456728 | 0.773 |
| **Cognitive function** |  |  |  |  |  |
| Logical memory immediately | 0.93792 | 0.002725 | 2 | 0.677308 | 0.489 |
| TMT-A-time | 0.822111 | 0.003742 | 12 | 0.351619 | 0.693 |
| TMT-B-time | 0.936962 | 0.003793 | 16 | 0.355535 | 0.674 |
| Logic memory delayed | 0.926378 | 0.002854 | 26 | 0.875594 | 0.481 |
| **DSB** | 0.923364 | 0.004674 | 80 | -0.43861 | 0.765 |
| **GAF** | 0.65563 | 0.004428 | 0 | -0.42256 | 0.795 |
| **Onset** | 0.269486 | 0.002933 | 0 | -0.12347 | 0.898 |

Note: PANSS, the Positive and Negative Syndrome Scale; HAMD, Hamilton depression scale; YMRS, Young mania rating scale; TMT, trail making test; DSB, digital symbol substitution; GAF, global assessment of functioning.

**Supplementary Table 4 Centrality, predictability, and expected influence of nodes in the dimension-level network of the transdiagnostic sample of the validation cohort.**

|  | **Strength** | **Closeness** | **Betweeness** | **Expected Influence** | **Predictability** |
| --- | --- | --- | --- | --- | --- |
| **PANSS** |  |  |  |  |  |
| Positive factor | 0.694337 | 0.004302 | 8 | 0.322851 | 0.765 |
| Negative factor | 0.732753 | 0.004391 | 56 | 0.139677 | 0.774 |
| Disorganized factor | 1.030958 | 0.004871 | 64 | 0.362931 | 0.688 |
| Excited factor | 0.923747 | 0.004697 | 60 | 0.876958 | 0.751 |
| Depressed factor | 0.828229 | 0.003728 | 4 | 0.759272 | 0.673 |
| **HAMD** |  |  |  |  |  |
| Anxiety | 0.681008 | 0.003682 | 8 | 0.625938 | 0.803 |
| Depression | 1.209522 | 0.004167 | 72 | 0.68233 | 0.601 |
| Insomnia | 0.455421 | 0.002819 | 0 | 0.455421 | 0.85 |
| Somatic | 0.691622 | 0.003461 | 28 | 0.691622 | 0.781 |
| **YMRS** |  |  |  |  |  |
| young_tot | 0.723103 | 0.004294 | 28 | 0.278676 | 0.743 |
| **Cognitive function** |  |  |  |  |  |
| Logical memory immediately | 0.743944 | 0.001953 | 0 | 0.743944 | 0.532 |
| Logical memory delayed | 0.817932 | 0.002038 | 30 | 0.684108 | 0.511 |
| TMT-A-time | 0.766834 | 0.003374 | 82 | 0.209198 | 0.998 |
| TMT-B-time | 0.496704 | 0.003052 | 0 | 0.464347 | 0.998 |
| DSB | 0.543641 | 0.004068 | 96 | -0.35224 | 0.885 |
| **PSP** | 0.633413 | 0.003736 | 0 | -0.54429 | 0.843 |
| **Onset** | 0.335854 | 0.003159 | 0 | 0.067093 | 0.94 |

Note: PANSS, the Positive and Negative Syndrome Scale; HAMD, Hamilton depression scale; YMRS, Young mania rating scale; TMT, trail making test; DSB, digital symbol substitution; GAF, global assessment of functioning.

**Supplementary Table 5 Centrality, predictability, and expected influence of nodes in the item-level network of the transdiagnostic sample in the discovery cohort.**

|  | **Strength** | **Closeness** | **Betweenness** | **Expected Influence** | **Predictability** |
| --- | --- | --- | --- | --- | --- |
| **PANSS** |  |  |  |  |  |
| P1 | 1.183203 | 0.000843 | 152 | 0.751016 | 0.552 |
| P3 | 0.742846 | 0.000793 | 18 | 0.703838 | 0.668 |
| P5 | 0.716521 | 0.000823 | 84 | 0.716521 | 0.737 |
| G9 | 0.875725 | 0.000777 | 0 | 0.792213 | 0.61 |
| N1 | 1.175714 | 0.000966 | 252 | 0.931414 | 0.469 |
| N2 | 0.972377 | 0.000907 | 44 | 0.930888 | 0.467 |
| N3 | 1.188916 | 0.000904 | 30 | 1.188916 | 0.474 |
| N4 | 0.979558 | 0.000829 | 34 | 0.843776 | 0.55 |
| N6 | 0.993731 | 0.000942 | 50 | 0.993731 | 0.516 |
| G7 | 0.862113 | 0.001038 | 414 | 0.862113 | 0.622 |
| P2 | 1.164464 | 0.00084 | 68 | 0.975983 | 0.601 |
| N5 | 0.934317 | 0.000896 | 146 | 0.806197 | 0.641 |
| G11 | 0.831969 | 0.000953 | 152 | 0.660939 | 0.713 |
| P4 | 1.11856 | 0.000867 | 94 | 0.981062 | 0.593 |
| P7 | 0.748562 | 0.000844 | 42 | 0.748562 | 0.718 |
| G8 | 0.979262 | 0.000865 | 48 | 0.713116 | 0.659 |
| G14 | 0.868619 | 0.000845 | 94 | 0.868619 | 0.738 |
| G2 | 0.72799 | 0.000781 | 80 | 0.72799 | 0.704 |
| G3 | 0.52042 | 0.000843 | 98 | 0.52042 | 0.75 |
| G6 | 1.181884 | 0.000956 | 310 | 1.127927 | 0.535 |
| **HAMD** |  |  |  |  |  |
| HAMD 9 | 0.600055 | 0.000883 | 116 | 0.600055 | 0.775 |
| HAMD 10 | 1.132555 | 0.000773 | 162 | 1.077649 | 0.622 |
| HAMD 11 | 1.081136 | 0.000727 | 136 | 1.081136 | 0.714 |
| HAMD 15 | 0.587939 | 0.000675 | 12 | 0.587939 | 0.807 |
| HAMD 17 | 0.377695 | 0.000735 | 8 | 0.228319 | 0.833 |
| HAMD 1 | 1.281592 | 0.000986 | 168 | 1.113997 | 0.546 |
| HAMD 2 | 1.050985 | 0.000888 | 128 | 0.989751 | 0.665 |
| HAMD 3 | 0.485049 | 0.000746 | 0 | 0.485049 | 0.836 |
| HAMD 7 | 0.945527 | 0.001054 | 504 | 0.767464 | 0.688 |
| HAMD 8 | 0.709236 | 0.001063 | 424 | 0.571738 | 0.717 |
| HAMD 4 | 0.699101 | 0.00072 | 114 | 0.699101 | 0.788 |
| HAMD 5 | 0.819729 | 0.00068 | 14 | 0.819729 | 0.77 |
| HAMD 6 | 0.560745 | 0.000619 | 4 | 0.502946 | 0.841 |
| HAMD 12 | 0.725731 | 0.000788 | 74 | 0.725731 | 0.77 |
| HAMD 13 | 0.633831 | 0.00073 | 46 | 0.633831 | 0.805 |
| HAMD 14 | 0.295043 | 0.000574 | 0 | 0.295043 | 0.893 |
| HAMD 16 | 0.405735 | 0.000703 | 0 | 0.405735 | 0.896 |
| **YMRS** | 0.829763 | 0.000794 | 50 | 0.746538 | 0.629 |
| **Cognition** |  |  |  |  |  |
| Logical memory immediately | 0.825771 | 0.00054 | 12 | 0.67751 | 0.504 |
| TMT-A-time | 0.643721 | 0.000608 | 0 | 0.357306 | 0.699 |
| TMT-B-time | 0.729623 | 0.000642 | 170 | 0.378024 | 0.684 |
| Logic memory delayed | 0.7753 | 0.000554 | 72 | 0.7753 | 0.512 |
| DSB | 0.707792 | 0.000768 | 330 | -0.44176 | 0.758 |
| **GAF** | 0.615726 | 0.000913 | 146 | -0.53511 | 0.753 |
| **Age Onset** | 0.086014 | 0.000423 | 0 | 0.020605 | 0.883 |

Note: PANSS, the Positive and Negative Syndrome Scale; P, positive factor; N, negative factor; G, general psychopathology; HAMD, Hamilton depression scale; YMRS, Young mania rating scale; TMT, trail making test; DSB, digital symbol substitution; GAF, global assessment of functioning.

**Supplementary Table 6 Centrality, predictability, and expected influence of nodes in the item-level network of the transdiagnostic sample in the validation cohort.**

|  | **Strength** | **Closeness** | **Betweenness** | **Expected Influence** | **Predictability** |
| --- | --- | --- | --- | --- | --- |
| **PANSS** |  |  |  |  |  |
| P1 | 0.914867 | 0.02943 | 264 | 0.778067 | 0.546 |
| P3 | 0.494512 | 0.029506 | 238 | 0.518486 | 0.439 |
| P5 | 0.753468 | 0.030263 | 230 | 0.764297 | 0.497 |
| G9 | 0.586171 | 0.027689 | 46 | 0.643643 | 0.386 |
| N1 | 1.098132 | 0.030892 | 282 | 1.007536 | 0.73 |
| N2 | 1.020761 | 0.029639 | 108 | 1.070954 | 0.75 |
| N3 | 0.848746 | 0.028792 | 0 | 0.824665 | 0.671 |
| N4 | 0.877446 | 0.028855 | 16 | 0.899631 | 0.671 |
| N6 | 0.826869 | 0.030026 | 122 | 0.887021 | 0.676 |
| G7 | 0.783457 | 0.030462 | 180 | 0.847585 | 0.566 |
| P2 | 0.883936 | 0.029273 | 400 | 0.711478 | 0.484 |
| N5 | 0.242853 | 0.028558 | 102 | 0.262934 | 0.299 |
| G11 | 0.307836 | 0.023629 | 12 | 0.383036 | 0.304 |
| P4 | 0.919852 | 0.030848 | 386 | 0.992635 | 0.651 |
| P7 | 0.700289 | 0.02692 | 48 | 0.759033 | 0.508 |
| G8 | 0.541643 | 0.025408 | 20 | 0.614335 | 0.464 |
| G14 | 0.656481 | 0.025992 | 14 | 0.704308 | 0.424 |
| G2 | 0.569361 | 0.029408 | 58 | 0.636061 | 0.528 |
| G3 | 0.611153 | 0.030513 | 122 | 0.674386 | 0.494 |
| G6 | 1.052187 | 0.030556 | 70 | 1.046688 | 0.687 |
| **HAMD** |  |  |  |  |  |
| HAMD 9 | 0.232453 | 0.029864 | 204 | 0.343406 | 0.324 |
| HAMD 10 | 0.751556 | 0.031114 | 222 | 0.806132 | 0.586 |
| HAMD 11 | 0.853154 | 0.028293 | 100 | 0.926624 | 0.504 |
| HAMD 15 | 0.043353 | 0.01386 | 0 | 0.101815 | 0.094 |
| HAMD 17 | 0.067977 | 0.018192 | 0 | -0.01296 | 0.174 |
| HAMD 1 | 1.14277 | 0.03202 | 504 | 0.864216 | 0.722 |
| HAMD 2 | 0.835997 | 0.031002 | 86 | 0.860235 | 0.593 |
| HAMD 3 | 0.758042 | 0.029 | 120 | 0.774344 | 0.606 |
| HAMD 7 | 0.565128 | 0.030987 | 278 | 0.623947 | 0.41 |
| HAMD 8 | 0.555282 | 0.030739 | 312 | 0.616323 | 0.466 |
| HAMD 4 | 0.67953 | 0.022848 | 110 | 0.77091 | 0.534 |
| HAMD 5 | 0.645904 | 0.022356 | 90 | 0.749123 | 0.519 |
| HAMD 6 | 0.452556 | 0.024561 | 202 | 0.522441 | 0.326 |
| HAMD 12 | 0.549087 | 0.028001 | 20 | 0.629231 | 0.369 |
| HAMD 13 | 0.442623 | 0.025541 | 0 | 0.514302 | 0.384 |
| HAMD 14 | 0.026457 | 0.012251 | 0 | 0.06135 | 0.096 |
| HAMD 16 | 0.095622 | 0.017011 | 0 | 0.160269 | 0.103 |
| YMRS |  |  |  |  |  |
| YMRS | 0.497514 | 0.029294 | 110 | 0.484369 | 0.559 |
| **Cognition** |  |  |  |  |  |
| Logical memory immediate | 0.497514 | 0.029294 | 0 | 0.614595 | 0.728 |
| Logical memory delayed | 0.497514 | 0.029294 | 0 | 0.533152 | 0.737 |
| TMT-A-time | 0.497514 | 0.029294 | 82 | 0.253901 | 0 |
| TMT-B-time | 0.497514 | 0.029294 | 0 | 0.397325 | 0 |
| DSB | 0.497514 | 0.029294 | 160 | -0.17942 | 0.075 |
| **PSP** | 0.257595 | 0.029294 | 0 | -0.31078 | 0.268 |
| **Age Onset** | 0.158377 | 0.029294 | 0 | -0.01154 | 0.136 |

Note: PANSS, the Positive and Negative Syndrome Scale; P, positive factor; N, negative factor; G, general psychopathology; HAMD, Hamilton depression scale; YMRS, Young mania rating scale; TMT, trail making test; DSB, digital symbol substitution; PSP, personal and social performance.

**Supplementation Table 7 Arc strength for the DAG in the dimension-level network of the transdiagnostic sample in the discovery cohort.**

| **Edge**  **From** | **To** | **Arc strength** | % of observed edge across bootstrap iterations | % of observed direction across bootstrap iterations |
| --- | --- | --- | --- | --- |
| PosF | Depression | -10.0269 | 0.754 | 0.758621 |
| NegF | PosF | -10.727 | 0.99 | 0.557071 |
| NegF | Depression | -4.22425 | 0.838 | 0.620525 |
| NegF | YMRS | -8.20938 | 0.946 | 0.643235 |
| DisF | PosF | -5.10017 | 0.897 | 0.758082 |
| DisF | NegF | -85.2351 | 1 | 0.688 |
| DisF | ExcF | -71.1582 | 1 | 0.7435 |
| DisF | DSB | -27.7654 | 0.911 | 0.7618 |
| ExcF | PosF | -13.3475 | 0.806 | 0.728908 |
| ExcF | Depression | -12.4097 | 0.793 | 0.759773 |
| ExcF | YMRS | -55.9413 | 1 | 0.8135 |
| DepF | Anxiety | -24.9306 | 0.999 | 0.82032 |
| DepF | Depression | -107.552 | 1 | 0.5045 |
| Depression | Somatic | -54.6629 | 1 | 0.6845 |
| Somatic | Anxiety | -16.0836 | 0.999 | 0.577077 |
| Somatic | Insomnia | -40.0505 | 0.989 | 0.827604 |
| YMRS | Anxiety | -16.5256 | 0.945 | 0.85291 |
| Logical memory immediately | Logic memory delayed | -227.596 | 1 | 0.5185 |
| TMT B time | ExcF | -7.71034 | 0.839 | 0.758641 |
| TMT B time | TMT A time | -101.486 | 1 | 0.5715 |
| TMT B time | DSB | -19.2356 | 0.888 | 0.503378 |
| GAF | Depression | -16.8854 | 0.985 | 0.600508 |
| Onset | DSB | -6.9331 | 0.918 | 0.658497 |

Note: PANSS, the Positive and Negative Syndrome Scale; PosF, positive factor; NegF, negative factor; DisF, disorganized factor; ExcF, excited factor; DepF, depressive factor; HAMD, Hamilton depression scale; YMRS, Young mania rating scale; TMT, trail making test; DSB, digital symbol substitution; GAF, global assessment of functioning.

**Supplementary Table 8 Arc strength for the DAG in the dimension-level network of the transdiagnostic sample in the validation cohort.**

| **Edge From** | **To** | **Arc strength** | % of observed edge across bootstrap iterations | % of observed direction across bootstrap iterations |
| --- | --- | --- | --- | --- |
| PosF | PSP | -17.1664 | 0.965 | 0.760104 |
| NegF | PSP | -6.84968 | 0.881 | 0.584563 |
| DisF | PosF | -39.3487 | 1 | 0.6105 |
| DisF | NegF | -31.3005 | 0.992 | 0.845766 |
| DisF | ExcF | -20.3791 | 0.91 | 0.714835 |
| DisF | DSB | -13.841 | 0.877 | 0.731471 |
| ExcF | YMRS | -25.8401 | 1 | 0.5155 |
| DepF | Anxiety | -9.36189 | 0.882 | 0.814059 |
| YMRS | NegF | -9.34083 | 0.905 | 0.58674 |
| Depression | DepF | -70.1895 | 1 | 0.619 |
| Depression | Somatic | -36.055 | 0.989 | 0.692113 |
| Somatic | Anxiety | -7.79937 | 0.906 | 0.586093 |
| Somatic | Insomnia | -23.9064 | 0.972 | 0.717593 |
| Logical memory immediately | Logical memory delayed | -146.148 | 0.212 | 0.566038 |
| DSB | TMTA_time | -11.7249 | 0.964 | 0.677386 |
| Onset | DSB | -17.1664 | 0.09 | 0.355556 |

Note: PANSS, the Positive and Negative Syndrome Scale; PosF, positive factor; NegF, negative factor; DisF, disorganized factor; ExcF, excited factor; DepF, depressive factor;HAMD, Hamilton depression scale; YMRS, Young mania rating scale; TMT, trail making test; DSB, digital symbol substitution; PSP, personal and social performance.

**Supplementary Table 9 Arc strength for the DAG in the item-level network of the discovery cohort.**

| **Edge**  **From** | **To** | **Arc strength** | % of observed edge across bootstrap iterations | % of observed direction across bootstrap iterations |
| --- | --- | --- | --- | --- |
| P1 | P3 | -112.876 | 1 | 0.708 |
| P1 | G9 | -123.788 | 1 | 0.635 |
| P1 | P2 | -37.1377 | 0.915 | 0.518579 |
| P1 | GAF | -37.0229 | 0.94 | 0.946809 |
| P5 | G3 | -14.7053 | 0.877 | 0.830103 |
| P5 | YMRS | -7.84911 | 0.972 | 0.525206 |
| N1 | N6 | -40.3584 | 0.978 | 0.526074 |
| N1 | G7 | -18.8243 | 0.98 | 0.8 |
| N1 | DSB | -30.8877 | 0.922 | 0.781996 |
| N2 | N1 | -165.636 | 1 | 0.541 |
| N2 | N3 | -157.816 | 0.98 | 0.537245 |
| N2 | N4 | -28.9261 | 0.998 | 0.701403 |
| N3 | N4 | -29.6871 | 0.969 | 0.711558 |
| N3 | N6 | -45.2966 | 0.999 | 0.527528 |
| N6 | G7 | -13.198 | 0.872 | 0.747133 |
| G7 | G11 | -6.95966 | 0.923 | 0.804984 |
| G7 | HAMD 8 | -27.7887 | 0.873 | 0.793814 |
| N5 | P2 | -62.8571 | 1 | 0.5245 |
| N5 | G11 | -10.4447 | 0.892 | 0.899664 |
| N5 | G14 | -21.1471 | 0.87 | 0.768966 |
| G11 | HAMD 9 | -23.9582 | 1 | 0.502 |
| P4 | P5 | -67.8311 | 0.989 | 0.766936 |
| P4 | G14 | -56.6577 | 0.996 | 0.766566 |
| P4 | YMRS | -40.2999 | 1 | 0.642 |
| G8 | G11 | -12.709 | 0.938 | 0.508529 |
| G8 | P7 | -68.435 | 1 | 0.532 |
| G6 | G2 | -4.66027 | 0.864 | 0.579282 |
| HAMD 10 | G2 | -50.96 | 1 | 0.5575 |
| HAMD 10 | HAMD 11 | -57.8926 | 0.972 | 0.694444 |
| HAMD 10 | HAMD 15 | -11.1466 | 0.966 | 0.889752 |
| HAMD 11 | HAMD 15 | -12.7355 | 0.916 | 0.742904 |
| HAMD 11 | HAMD 13 | -29.9939 | 0.942 | 0.768577 |
| HAMD 1 | G6 | -155.95 | 1 | 0.5205 |
| HAMD 1 | HAMD 2 | -57.5011 | 0.96 | 0.795833 |
| HAMD 2 | G3 | -48.547 | 1 | 0.656 |
| HAMD 7 | HAMD 8 | -24.8829 | 0.977 | 0.570113 |
| HAMD 7 | GAF | -20.7714 | 0.996 | 0.753012 |
| HAMD 4 | HAMD 5 | -47.1006 | 1 | 0.532 |
| HAMD 4 | HAMD 6 | -25.1591 | 0.952 | 0.75105 |
| HAMD 13 | HAMD 12 | -30.8714 | 0.964 | 0.538382 |
| YMRS | HAMD 17 | -20.9836 | 0.999 | 0.582082 |
| Logical memory immed | Logic memory delayed | -217.958 | 1 | 0.5085 |
| TMT-B-time | TMT-A-time | -93.447 | 1 | 0.5165 |
| Onset | DSB | -9.78837 | 0.949 | 0.689146 |

Note: PANSS, the Positive and Negative Syndrome Scale; P, positive factor; N, negative factor; G, general psychopathology; HAMD, Hamilton depression scale; YMRS, Young mania rating scale; TMT, trail making test; DSB, digital symbol substitution; GAF, global assessment of functioning.

**Supplementary Table 10 Arc strength for the DAG in the item-level network of the validation cohort.**

| **Edge**  **From** | **To** | **Arc strength** | % of observed edge across bootstrap iterations | % of observed direction across bootstrap iterations |
| --- | --- | --- | --- | --- |
| P1 | P3 | -39.922 | 1 | 0.5395 |
| P1 | G9 | -36.5557 | 0.996 | 0.823795 |
| P1 | P2 | -36.2423 | 0.97 | 0.731443 |
| N1 | P3 | -12.1987 | 0.918 | 0.689542 |
| N1 | N3 | -31.8581 | 0.995 | 0.598995 |
| N1 | G7 | -79.0917 | 1 | 0.841 |
| N2 | N3 | -18.8921 | 0.929 | 0.62648 |
| N2 | N4 | -103.648 | 1 | 0.5055 |
| N6 | N5 | -23.0461 | 0.943 | 0.883351 |
| N6 | HAMD8 | -31.7225 | 0.861 | 0.71777 |
| P4 | P5 | -17.0782 | 0.979 | 0.668539 |
| P4 | P7 | -29.1544 | 0.914 | 0.628556 |
| P4 | YMRS | -51.8086 | 0.999 | 0.697197 |
| P7 | G8 | -35.3821 | 0.996 | 0.75 |
| P7 | G14 | -48.6701 | 0.997 | 0.573721 |
| G3 | N2 | -13.6918 | 0.886 | 0.716704 |
| G3 | G2 | -15.1763 | 0.934 | 0.661135 |
| G6 | G7 | -8.43655 | 0.862 | 0.781323 |
| G6 | HAMD1 | -96.8278 | 1 | 0.5035 |
| G6 | HAMD3 | -63.4216 | 0.964 | 0.748963 |
| YMRS | P5 | -4.00986 | 0.858 | 0.567016 |
| HAMD10 | G2 | -23.9865 | 1 | 0.6015 |
| HAMD10 | HAMD9 | -8.04983 | 0.921 | 0.692725 |
| HAMD11 | HAMD13 | -46.3171 | 0.999 | 0.596096 |
| HAMD1 | HAMD2 | -42.3618 | 0.967 | 0.708893 |
| HAMD1 | HAMD7 | -36.5516 | 0.902 | 0.882483 |
| HAMD2 | G3 | -55.5727 | 1 | 0.6185 |
| HAMD7 | HAMD9 | 0.301089 | 0.855 | 0.68655 |
| HAMD4 | HAMD5 | -63.4897 | 1 | 0.533 |
| HAMD5 | HAMD6 | -29.5168 | 0.963 | 0.748183 |
| Logical memory delayed | Logical memory immediate | -153.638 | 1 | 0.501 |
| TMT-A-time | TMT-B-time | -77.0603 | 1 | 0.5865 |
| TMT-A-time | DSB | -13.0445 | 0.969 | 0.71001 |

Note: PANSS, the Positive and Negative Syndrome Scale; P, positive factor; N, negative factor; G, general psychopathology; HAMD, Hamilton depression scale; YMRS, Young mania rating scale; TMT, trail making test; DSB, digital symbol substitution; PSP, personal and social performance.

**Supplementary Table 11 Gender Network comparison of the dimension level in the discovery cohort.**

| **Variable 1** | **Variable 2** | **p.value** | **Test.statistic.E** |
| --- | --- | --- | --- |
| PosF | NegF | 1 | 0.0017238 |
| PosF | DisF | 1 | 0.07117464 |
| NegF | DisF | 1 | 0.04035825 |
| PosF | ExcF | 1 | 0.06687448 |
| NegF | ExcF | 1 | 0 |
| DisF | ExcF | 1 | 0.07268735 |
| PosF | DepF | 1 | 0 |
| NegF | DepF | 1 | 0.05270077 |
| DisF | DepF | 1 | 0 |
| ExcF | DepF | 1 | 0 |
| PosF | Anxiety | 1 | 0 |
| NegF | Anxiety | 1 | 0 |
| DisF | Anxiety | 1 | 0.03858267 |
| ExcF | Anxiety | 1 | 0.06676686 |
| DepF | Anxiety | 1 | 0.05474809 |
| PosF | Depression | 1 | 0 |
| NegF | Depression | 1 | 0.00668246 |
| DisF | Depression | 1 | 0 |
| ExcF | Depression | 1 | 0.00973837 |
| DepF | Depression | 1 | 0.03711633 |
| Anxiety | Depression | 1 | 0 |
| PosF | Insomnia | 1 | 0 |
| NegF | Insomnia | 1 | 0 |
| DisF | Insomnia | 1 | 0 |
| ExcF | Insomnia | 1 | 0 |
| DepF | Insomnia | 1 | 0.1458871 |
| Anxiety | Insomnia | 1 | 0.01229872 |
| Depression | Insomnia | 1 | 0.02704784 |
| PosF | Somatic | 1 | 0 |
| NegF | Somatic | 1 | 0 |
| DisF | Somatic | 1 | 0 |
| ExcF | Somatic | 1 | 0 |
| DepF | Somatic | 1 | 0.04541104 |
| Anxiety | Somatic | 1 | 0.09343105 |
| Depression | Somatic | 1 | 0.06066899 |
| Insomnia | Somatic | 1 | 0.01217785 |
| PosF | YMRS | 1 | 0.0484897 |
| NegF | YMRS | 1 | 0 |
| DisF | YMRS | 1 | 0 |
| ExcF | YMRS | 1 | 0.09010048 |
| DepF | YMRS | 1 | 0 |
| Anxiety | YMRS | 1 | 0.14168765 |
| Depression | YMRS | 1 | 0.02465162 |
| Insomnia | YMRS | 1 | 0 |
| Somatic | YMRS | 1 | 0 |
| PosF | Logical memory immedately | 1 | 0 |
| NegF | Logical memory immedately | 1 | 0 |
| DisF | Logical memory immedately | 1 | 0 |
| ExcF | Logical memory immedately | 1 | 0 |
| DepF | Logical memory immedately | 1 | 0 |
| Anxiety | Logical memory immedately | 1 | 0 |
| Depression | Logical memory immedately | 1 | 0.02207877 |
| Insomnia | Logical memory immedately | 1 | 0 |
| Somatic | Logical memory immedately | 1 | 0.03883155 |
| YMRS | Logical memory immedately | 1 | 0 |
| PosF | TMT-A-time | 1 | 0 |
| NegF | TMT-A-time | 1 | 0 |
| DisF | TMT-A-time | 1 | 0 |
| ExcF | TMT-A-time | 1 | 0 |
| DepF | TMT-A-time | 1 | 0.02236771 |
| Anxiety | TMT-A-time | 1 | 0 |
| Depression | TMT-A-time | 1 | 0 |
| Insomnia | TMT-A-time | 1 | 0 |
| Somatic | TMT-A-time | 1 | 0 |
| YMRS | TMT-A-time | 1 | 0 |
| Logical memory immediately | TMT-A-time | 1 | 0.01283674 |
| PosF | TMT-B-time | 1 | 0 |
| NegF | TMT-B-time | 0.135986401359864 | 0.10350423 |
| DisF | TMT-B-time | 1 | 0 |
| ExcF | TMT-B-time | 1 | 0 |
| DepF | TMT-B-time | 1 | 0 |
| Anxiety | TMT-B-time | 1 | 0 |
| Depression | TMT-B-time | 1 | 0 |
| Insomnia | TMT-B-time | 1 | 0 |
| Somatic | TMT-B-time | 1 | 0 |
| young.tot | TMT-B-time | 1 | 0 |
| Logical memory immediately | TMT-B-time | 1 | 0.03794202 |
| TMT-A-time | TMT-B-time | 1 | 0.00624594 |
| PosF | Logiccal memory delayed | 1 | 0.01916124 |
| NegF | Logiccal memory delayed | 1 | 0 |
| DisF | Logiccal memory delayed | 1 | 0 |
| ExcF | Logiccal memory delayed | 1 | 0 |
| DepF | Logiccal memory delayed | 1 | 0.02086863 |
| Anxiety | Logiccal memory delayed | 1 | 0 |
| Depression | Logiccal memory delayed | 1 | 0 |
| Insomnia | Logiccal memory delayed | 1 | 0 |
| Somatic | Logiccal memory delayed | 1 | 0.01209989 |
| young.tot | Logiccal memory delayed | 1 | 0 |
| Logical memory immediately | Logiccal memory delayed | 1 | 0.05953521 |
| TMT-A-time | Logiccal memory delayed | 1 | 0 |
| TMT-B-time | Logiccal memory delayed | 1 | 0 |
| PosF | DSB | 1 | 0.03541093 |
| NegF | DSB | 1 | 0.12516317 |
| DisF | DSB | 1 | 0.1755071 |
| ExcF | DSB | 1 | 0 |
| DepF | DSB | 1 | 0 |
| Anxiety | DSB | 1 | 0 |
| Depression | DSB | 1 | 0 |
| Insomnia | DSB | 1 | 0 |
| Somatic | DSB | 1 | 0 |
| YMRS | DSB | 1 | 0 |
| Logical memory immedately | DSB | 0.736926307369263 | 0.12334935 |
| TMT-A-time | DSB | 1 | 0.03892363 |
| TMT-B-time | DSB | 1 | 0.07584203 |
| Logical memory delayed | DSB | 1 | 0.02381831 |
| PosF | GAF | 1 | 0.03420927 |
| NegF | GAF | 1 | 0.13219671 |
| DisF | GAF | 1 | 0.04024746 |
| ExcF | GAF | 1 | 0.04721605 |
| DepF | GAF | 1 | 0 |
| Anxiety | GAF | 1 | 0 |
| Depression | GAF | 1 | 0.0780658 |
| Insomnia | GAF | 1 | 0 |
| Somatic | GAF | 1 | 0 |
| young.tot | GAF | 1 | 0 |
| Logical memory immediately | GAF | 1 | 0 |
| TMT-A-time | GAF | 1 | 0 |
| TMT-B-time | GAF | 1 | 0 |
| Logical memory delayed | GAF | 1 | 0 |
| DSB | GAF | 1 | 0.0124345 |
| PosF | Onset | 1 | 0 |
| NegF | Onset | 1 | 0 |
| DisF | Onset | 1 | 0 |
| ExcF | Onset | 1 | 0.01945279 |
| DepF | Onset | 1 | 0 |
| Anxiety | Onset | 1 | 0 |
| Depression | Onset | 1 | 0 |
| Insomnia | Onset | 0.445455454454555 | 0.03648465 |
| Somatic | Onset | 1 | 0.02960214 |
| young.tot | Onset | 1 | 0 |
| Logical memory immediately | Onset | 1 | 0 |
| TMT-A-time | Onset | 1 | 0 |
| TMT.B.time | Onset | 1 | 0.04233331 |
| Logical memory delayed | Onset | 1 | 0.02559962 |
| DSB | Onset | 1 | 0.12535436 |
| GAF | Onset | 1 | 0 |

Note: PANSS, the Positive and Negative Syndrome Scale; PosF, positive factor; NegF, negative factor; DisF, disorganized factor; ExcF, excited factor; DepF, depressive factor; HAMD, Hamilton depression scale; YMRS, Young mania rating scale; TMT, trail making test; DSB, digital symbol substitution; GAF, global assessment of functioning.

**Supplementary Table12 Disease network comparison of the dimensional level in the discovery cohort**

| **Variable 1** | **Variable 2** | **p.value** | **Test.statistic.E** |
| --- | --- | --- | --- |
| PosF | NegF | 1 | 0.0386509 |
| PosF | DisF | 1 | 0.17750098 |
| NegF | DisF | 1 | 0.13548514 |
| PosF | ExcF | 0.0916908309169083 | 0.24647575 |
| NegF | ExcF | 0.0535946405359464 | 0.11113591 |
| DisF | ExcF | 1 | 0.16799045 |
| PosF | DepF | 1 | 0.02778172 |
| NegF | DepF | 0.0535946405359464 | 0.17763006 |
| DisF | DepF | 1 | 0 |
| ExcF | DepF | 1 | 0 |
| PosF | Anxiety | 1 | 0 |
| NegF | Anxiety | 1 | 0 |
| DisF | Anxiety | 1 | 0.00955184 |
| ExcF | Anxiety | 1 | 0.11410616 |
| DepF | Anxiety | 1 | 0.02891671 |
| PosF | Depression | 1 | 0 |
| NegF | Depression | 1 | 0.00647868 |
| DisF | Depression | 1 | 0 |
| ExcF | Depression | 1 | 0.06049215 |
| DepF | Depression | 1 | 0.20127398 |
| Anxiety | Depression | 1 | 0.05338166 |
| PosF | Insomnia | 0.0135986401359864 | 0.10676524 |
| NegF | Insomnia | 1 | 0 |
| DisF | Insomnia | 1 | 0 |
| ExcF | Insomnia | 1 | 0 |
| DepF | Insomnia | 1 | 0.13190469 |
| Anxiety | Insomnia | 0.0916908309169083 | 0.27004299 |
| Depression | Insomnia | 1 | 0.04221309 |
| PosF | Somatic | 1 | 0 |
| NegF | Somatic | 1 | 0.04898074 |
| DisF | Somatic | 1 | 0 |
| ExcF | Somatic | 1 | 0 |
| DepF | Somatic | 0.0535946405359464 | 0.14788742 |
| Anxiety | Somatic | 1 | 0.11160452 |
| Depression | Somatic | 1 | 0.01036604 |
| Insomnia | Somatic | 1 | 0.10969858 |
| PosF | YMRS | 0.0135986401359864 | 0.23158017 |
| NegF | YMRS | 1 | 0.02113099 |
| DisF | YMRS | 1 | 0 |
| ExcF | YMRS | 1 | 0.21315954 |
| DepF | YMRS | 1 | 0.03018504 |
| Anxiety | YMRS | 1 | 0.00569699 |
| Depression | YMRS | 0.952404759524048 | 0.15670499 |
| Insomnia | YMRS | 1 | 0 |
| Somatic | YMRS | 1 | 0 |
| PosF | Logical memory immediately | 1 | 0 |
| NegF | Logical memory immediately | 1 | 0 |
| DisF | Logical memory immediately | 1 | 0.0030632 |
| ExcF | Logical memory immediately | 1 | 0 |
| DepF | Logical memory immediately | 1 | 0 |
| Anxiety | Logical memory immediately | 1 | 0 |
| Depression | Logical memory immediately | 1 | 0.05524989 |
| Insomnia | Logical memory immediately | 1 | 0 |
| Somatic | Logical memory immediately | 1 | 0 |
| YMRS | Logical memory immediately | 1 | 0.02633271 |
| PosF | TMT-A-time | 1 | 0 |
| NegF | TMT-A-time | 1 | 0 |
| DisF | TMT-A-time | 1 | 0 |
| ExcF | TMT-A-time | 1 | 0 |
| DepF | TMT-A-time | 1 | 0.03354228 |
| Anxiety | TMT-A-time | 1 | 0 |
| Depression | TMT-A-time | 1 | 0.04304374 |
| Insomnia | TMT-A-time | 1 | 0 |
| Somatic | TMT-A-time | 1 | 0 |
| young.tot | TMT-A-time | 1 | 0.04676911 |
| Logical memory immediately | TMT-A-time | 1 | 0.03987047 |
| PosF | TMT-B-time | 1 | 0 |
| NegF | TMT-B-time | 1 | 0 |
| DisF | TMT-B-time | 1 | 0 |
| ExcF | TMT-B-time | 1 | 0.02222074 |
| DepF | TMT-B-time | 1 | 0 |
| Anxiety | TMT-B-time | 1 | 0 |
| Depression | TMT-B-time | 1 | 0 |
| Insomnia | TMT-B-time | 1 | 0 |
| Somatic | TMT-B-time | 1 | 0 |
| YMRS | TMT-B-time | 1 | 0 |
| Logical memory immediately | TMT-B-time | 1 | 0.02239041 |
| TMT-A-time | TMT-B-time | 1 | 0.11408871 |
| PosF | Logical memory delayed | 1 | 0 |
| NegF | Logical memory delayed | 1 | 0 |
| DisF | Logical memory delayed | 1 | 0.04533714 |
| ExcF | Logical memory delayed | 1 | 0 |
| DepF | Logical memory delayed | 1 | 0.07673875 |
| Anxiety | Logical memory delayed | 1 | 0 |
| Depression | Logical memory delayed | 1 | 0 |
| Insomnia | Logical memory delayed | 1 | 0 |
| Somatic | Logical memory delayed | 1 | 0.0517305 |
| YMRS | Logical memory delayed | 1 | 0 |
| Logical memory delayed | Logical memory delayed | 1 | 0.01229373 |
| TMT-A-time | Logical memory delayed | 1 | 0.04458094 |
| TMT-B-time | Logical memory delayed | 1 | 0.01486889 |
| PosF | DSB | 1 | 0.0084312 |
| NegF | DSB | 1 | 0.00724426 |
| DisF | DSB | 1 | 0.01736629 |
| ExcF | DSB | 1 | 0 |
| DepF | DSB | 1 | 0 |
| Anxiety | DSB | 1 | 0 |
| Depression | DSB | 1 | 0 |
| Insomnia | DSB | 1 | 0 |
| Somatic | DSB | 1 | 0 |
| young.tot | DSB | 1 | 0.02501918 |
| Logical memory immediately | DSB | 1 | 0.07102804 |
| TMT-A-time | DSB | 1 | 0.08319704 |
| TMT-B-time | DSB | 1 | 0.04847543 |
| Logical memory delayed | DSB | 1 | 0.07901558 |
| PosF | GAF | 1 | 0.17247182 |
| NegF | GAF | 1 | 0.03171731 |
| DisF | GAF | 1 | 0.03536978 |
| ExcF | GAF | 1 | 0.01292786 |
| DepF | GAF | 1 | 0 |
| Anxiety | GAF | 1 | 0 |
| Depression | GAF | 0.0916908309169083 | 0.21098955 |
| Insomnia | GAF | 1 | 0.03341865 |
| Somatic | GAF | 1 | 0 |
| young.tot | GAF | 1 | 0.02693621 |
| Logical memory immediately | GAF | 1 | 0.02562157 |
| TMT-A-time | GAF | 0.345565443455654 | 0.06046934 |
| TMT-B-time | GAF | 1 | 0 |
| Logical memory delayed | GAF | 1 | 0 |
| DSB | GAF | 1 | 0.02345706 |
| PosF | Onset | 1 | 0 |
| NegF | Onset | 1 | 0 |
| DisF | Onset | 1 | 0.03445777 |
| ExcF | Onset | 1 | 0.08368436 |
| DepF | Onset | 1 | 0.00625187 |
| Anxiety | Onset | 1 | 0 |
| Depression | Onset | 1 | 0 |
| Insomnia | Onset | 1 | 0 |
| Somatic | Onset | 1 | 0 |
| young.tot | Onset | 1 | 0.00938121 |
| Logical memory immediately | Onset | 1 | 0 |
| TMT-A-time | Onset | 1 | 0 |
| TMT-B-time | Onset | 1 | 0.11195761 |
| Logical memory delayed | Onset | 1 | 0.06281983 |
| DSB | Onset | 1 | 0.08869778 |
| GAF | Onset | 1 | 0 |

Note: PANSS, the Positive and Negative Syndrome Scale; PosF, positive factor; NegF, negative factor; DisF, disorganized factor; ExcF, excited factor; DepF, depressive factor; HAMD, Hamilton depression scale; YMRS, Young mania rating scale; TMT, trail making test; DSB, digital symbol substitution; GAF, global assessment of functioning.

**Supplementary Table 13 Gender network comparison of the dimension level in the validation cohort.**

| **Variable 1** | **Variable 2** | **p.value** | **Test.statistic.E** |
| --- | --- | --- | --- |
| PosF | NegF | 1 | 0.11528998 |
| PosF | DisF | 1 | 0.07088807 |
| NegF | DisF | 1 | 0.13497817 |
| PosF | ExcF | 1 | 0.12987785 |
| NegF | ExcF | 1 | 0 |
| DisF | ExcF | 1 | 0.1243046 |
| PosF | DepF | 0.308169183081692 | 0.0468605 |
| NegF | DepF | 1 | 0.02924653 |
| DisF | DepF | 1 | 0 |
| ExcF | DepF | 1 | 0 |
| PosF | YMRS | 1 | 0 |
| NegF | YMRS | 1 | 0.08151349 |
| DisF | YMRS | 1 | 0 |
| ExcF | YMRS | 1 | 0.35410523 |
| DepF | YMRS | 1 | 0 |
| PosF | Anxiety | 1 | 0 |
| NegF | Anxiety | 1 | 0 |
| DisF | Anxiety | 1 | 0 |
| ExcF | Anxiety | 1 | 0.11664098 |
| DepF | Anxiety | 1 | 0.067266 |
| YMRS | Anxiety | 1 | 0.13720915 |
| PosF | Depression | 1 | 0 |
| NegF | Depression | 1 | 0.09713473 |
| DisF | Depression | 1 | 0 |
| ExcF | Depression | 1 | 0 |
| DepF | Depression | 1 | 0.00992628 |
| YMRS | Depression | 0.598440155984401 | 0.16204569 |
| Anxiety | Depression | 1 | 0.11161935 |
| PosF | Insomnia | 1 | 0 |
| NegF | Insomnia | 1 | 0 |
| DisF | Insomnia | 1 | 0 |
| ExcF | Insomnia | 1 | 0 |
| DepF | Insomnia | 1 | 0.0587923 |
| YMRS | Insomnia | 1 | 0 |
| Anxiety | Insomnia | 1 | 0.07708173 |
| Depression | Insomnia | 1 | 0.08525396 |
| PosF | Somatic | 1 | 0 |
| NegF | Somatic | 1 | 0 |
| DisF | Somatic | 1 | 0 |
| ExcF | Somatic | 1 | 0 |
| DepF | Somatic | 1 | 0.00971056 |
| YMRS | Somatic | 1 | 0 |
| Anxiety | Somatic | 1 | 0.08712044 |
| Depression | Somatic | 1 | 0.13716986 |
| Insomnia | Somatic | 1 | 0.06042565 |
| PosF | Logical memory immediately | 1 | 0 |
| NegF | Logical memory immediately | 1 | 0 |
| DisF | Logical memory immediately | 1 | 0.00440692 |
| ExcF | Logical memory immediately | 1 | 0 |
| DepF | Logical memory immediately | 1 | 0 |
| YMRS | Logical memory immediately | 1 | 0 |
| Anxiety | Logical memory immediately | 1 | 0 |
| Depression | Logical memory immediately | 1 | 0 |
| Insomnia | Logical memory immediately | 1 | 0 |
| Somatic | Logical memory immediately | 1 | 0 |
| PosF | Logical memory delayed | 1 | 0 |
| NegF | Logical memory delayed | 1 | 0 |
| DisF | Logical memory delayed | 1 | 0 |
| ExcF | Logical memory delayed | 1 | 0 |
| DepF | Logical memory delayed | 1 | 0 |
| YMRS | Logical memory delayed | 1 | 0 |
| Anxiety | Logical memory delayed | 1 | 0 |
| Depression | Logical memory delayed | 1 | 0 |
| Insomnia | Logical memory delayed | 1 | 0 |
| Somatic | Logical memory delayed | 1 | 0.02317232 |
| Logical memory immediately | Logical memory delayed | 1 | 0.18503354 |
| PosF | TMT-A-time | 1 | 0 |
| NegF | TMT-A-time | 1 | 0 |
| DisF | TMT-A-time | 1 | 0 |
| ExcF | TMT-A-time | 1 | 0 |
| DepF | TMT-A-time | 1 | 0 |
| YMRS | TMT-A-time | 1 | 0 |
| Anxiety | TMT-A-time | 1 | 0 |
| Depression | TMT-A-time | 1 | 0 |
| Insomnia | TMT-A-time | 1 | 0 |
| Somatic | TMT-A-time | 1 | 0 |
| Logical memory immediately | TMT-A-time | 1 | 0 |
| Logical memory delayed | TMT-A-time | 1 | 0 |
| PosF | TMT-B-time | 1 | 0 |
| NegF | TMT-B-time | 1 | 0 |
| DisF | TMT-B-time | 1 | 0 |
| ExcF | TMT-B-time | 1 | 0.04253023 |
| DepF | TMT-B-time | 1 | 0 |
| YMRS | TMT-B-time | 1 | 0 |
| Anxiety | TMT-B-time | 1 | 0 |
| Depression | TMT-B-time | 1 | 0 |
| Insomnia | TMT-B-time | 0.0407959204079592 | 0.06601815 |
| Somatic | TMT-B-time | 1 | 0 |
| Logical memory immediately | TMT-B-time | 1 | 0 |
| Logical memory delayed | TMT-B-time | 1 | 0 |
| TMT-A-time | TMT-B-time | 1 | 0.35262363 |
| PosF | DSB | 1 | 0.03027486 |
| NegF | DSB | 1 | 0.01990175 |
| DisF | DSB | 1 | 0.07271102 |
| ExcF | DSB | 1 | 0 |
| DepF | DSB | 0.188981101889811 | 0.02310246 |
| YMRS | DSB | 1 | 0 |
| Anxiety | DSB | 1 | 0 |
| Depression | DSB | 1 | 0 |
| Insomnia | DSB | 1 | 0 |
| Somatic | DSB | 1 | 0 |
| Logical memory immediately | DSB | 1 | 0.10017185 |
| Logical memory delayed | DSB | 1 | 0 |
| TMT-A-time | DSB | 1 | 0.08033489 |
| TMT-B-time | DSB | 1 | 0 |
| PosF | PSP | 1 | 0.08381792 |
| NegF | PSP | 1 | 0.01091196 |
| DisF | PSP | 1 | 0.1542294 |
| ExcF | PSP | 1 | 0.05237586 |
| DepF | PSP | 1 | 0 |
| YMRS | PSP | 1 | 0.10030373 |
| Anxiety | PSP | 1 | 0.05300326 |
| Depression | PSP | 1 | 0 |
| Insomnia | PSP | 1 | 0 |
| Somatic | PSP | 1 | 0 |
| Logical memory immediately | PSP | 1 | 0 |
| Logical memory delayed | PSP | 1 | 0 |
| TMT-A-time | PSP | 1 | 0 |
| TMT-B-time | PSP | 1 | 0 |
| DSB | PSP | 1 | 0.00904457 |
| PosF | Onsetage | 1 | 0 |
| NegF | Onsetage | 1 | 0.03920817 |
| DisF | Onsetage | 1 | 0 |
| ExcF | Onsetage | 1 | 0 |
| DepF | Onsetage | 1 | 0 |
| YMRS | Onsetage | 1 | 0.05613126 |
| Anxiety | Onsetage | 1 | 0 |
| Depression | Onsetage | 1 | 0.04762452 |
| Insomnia | Onsetage | 1 | 0 |
| Somatic | Onsetage | 1 | 0 |
| Logical memory immediately | Onsetage | 1 | 0 |
| Logical memory delayed | Onsetage | 1 | 0 |
| TMT-A-time | Onsetage | 1 | 0 |
| TMT-B-time | Onsetage | 1 | 0 |
| DSB | Onsetage | 1 | 0.06853579 |
| PSP | Onsetage | 1 | 0 |

Note: PANSS, the Positive and Negative Syndrome Scale; PosF, positive factor; NegF, negative factor; DisF, disorganized factor; ExcF, excited factor; DepF, depressive factor;HAMD, Hamilton depression scale; YMRS, Young mania rating scale; TMT, trail making test; DSB, digital symbol substitution; PSP, personal and social performance.

**Supplementary Table 14 Disease network comparison of the dimension level in the validation cohort.**

| **Variable 1** | **Variable 2** | **p.value** | **Test.statistic.E** |
| --- | --- | --- | --- |
| PosF | NegF | 1 | 0 |
| PosF | DisF | 1 | 0.11534964 |
| NegF | DisF | 1 | 0.09712128 |
| PosF | ExcF | 1 | 0.08687017 |
| NegF | ExcF | 1 | 0 |
| DisF | ExcF | 1 | 0.03043842 |
| PosF | DepF | 1 | 0.02218339 |
| NegF | DepF | 1 | 0.08173057 |
| DisF | DepF | 1 | 0 |
| ExcF | DepF | 1 | 0.01082397 |
| PosF | YMRS | 0.0135986401359864 | 0.19925175 |
| NegF | YMRS | 1 | 0 |
| DisF | YMRS | 0.458454154584542 | 0.02172562 |
| ExcF | YMRS | 1 | 0.11595905 |
| DepF | YMRS | 1 | 0 |
| PosF | Anxiety | 1 | 0 |
| NegF | Anxiety | 1 | 0 |
| DisF | Anxiety | 1 | 0.05374335 |
| ExcF | Anxiety | 1 | 0.03717474 |
| DepF | Anxiety | 1 | 0.05217785 |
| YMRS | Anxiety | 1 | 0.07932965 |
| PosF | Depression | 1 | 0.01346896 |
| NegF | Depression | 1 | 0.1382142 |
| DisF | Depression | 1 | 0 |
| ExcF | Depression | 1 | 0 |
| DepF | Depression | 1 | 0.05143947 |
| YMRS | Depression | 0.0944905509449055 | 0.19679755 |
| Anxiety | Depression | 1 | 0.03988859 |
| PosF | Insomnia | 1 | 0 |
| NegF | Insomnia | 1 | 0 |
| DisF | Insomnia | 1 | 0 |
| ExcF | Insomnia | 1 | 0 |
| DepF | Insomnia | 1 | 0.02025828 |
| YMRS | Insomnia | 1 | 0 |
| Anxiety | Insomnia | 1 | 0.08040961 |
| Depression | Insomnia | 1 | 0.10958149 |
| PosF | Somatic | 1 | 0 |
| NegF | Somatic | 1 | 0 |
| DisF | Somatic | 1 | 0 |
| ExcF | Somatic | 1 | 0 |
| DepF | Somatic | 1 | 0.0304319 |
| YMRS | Somatic | 1 | 0 |
| Anxiety | Somatic | 1 | 0.01899549 |
| Depression | Somatic | 1 | 0.04638416 |
| Insomnia | Somatic | 1 | 0.03213167 |
| PosF | Logical memory immediately | 1 | 0 |
| NegF | Logical memory immediately | 1 | 0 |
| DisF | Logical memory immediately | 1 | 0 |
| ExcF | Logical memory immediately | 1 | 0 |
| DepF | Logical memory immediately | 1 | 0 |
| YMRS | Logical memory immediately | 0.395960403959604 | 0.03300302 |
| Anxiety | Logical memory immediately | 1 | 0 |
| Depression | Logical memory immediately | 1 | 0 |
| Insomnia | Logical memory immediately | 1 | 0 |
| Somatic | Logical memory immediately | 1 | 0 |
| PosF | Logical memory delayed | 1 | 0 |
| NegF | Logical memory delayed | 1 | 0 |
| DisF | Logical memory delayed | 1 | 0 |
| ExcF | Logical memory delayed | 0.200979902009799 | 0.01666522 |
| DepF | Logical memory delayed | 1 | 0 |
| YMRS | Logical memory delayed | 1 | 0 |
| Anxiety | Logical memory delayed | 1 | 0 |
| Depression | Logical memory delayed | 1 | 0 |
| Insomnia | Logical memory delayed | 1 | 0 |
| Somatic | Logical memory delayed | 1 | 0 |
| Logical memory immediately | Logical memory delayed | 1 | 0.00865224 |
| PosF | TMT-A-time | 1 | 0 |
| NegF | TMT-A-time | 1 | 0 |
| DisF | TMT-A-time | 1 | 0 |
| ExcF | TMT-A-time | 1 | 0 |
| DepF | TMT-A-time | 1 | 0 |
| YMRS | TMT-A-time | 1 | 0 |
| Anxiety | TMT-A-time | 1 | 0 |
| Depression | TMT-A-time | 1 | 0 |
| Insomnia | TMT-A-time | 1 | 0.0176852 |
| Somatic | TMT-A-time | 1 | 0 |
| Logical memory immediately | TMT-A-time | 1 | 0 |
| Logical memory delayed | TMT-A-time | 1 | 0 |
| PosF | TMT-B-time | 1 | 0 |
| NegF | TMT-B-time | 1 | 0 |
| DisF | TMT-B-time | 1 | 0 |
| ExcF | TMT-B-time | 1 | 0 |
| DepF | TMT-B-time | 1 | 0 |
| YMRS | TMT-B-time | 1 | 0 |
| Anxiety | TMT-B-time | 1 | 0 |
| Depression | TMT-B-time | 1 | 0 |
| Insomnia | TMT-B-time | 1 | 0 |
| Somatic | TMT-B-time | 1 | 0 |
| Logical memory immediately | TMT-B-time | 1 | 0 |
| Logical memory delayed | TMT-B-time | 1 | 0 |
| TMT-A-time | TMT-B-time | 0.279272072792721 | 0.59009353 |
| PosF | DSB | 1 | 0 |
| NegF | DSB | 1 | 0 |
| DisF | DSB | 1 | 0.01990858 |
| ExcF | DSB | 0.493950604939506 | 0.1007076 |
| DepF | DSB | 1 | 0 |
| YMRS | DSB | 1 | 0 |
| Anxiety | DSB | 1 | 0 |
| Depression | DSB | 1 | 0 |
| Insomnia | DSB | 1 | 0 |
| Somatic | DSB | 1 | 0 |
| Logical memory immediately | DSB | 1 | 0 |
| Logical memory delayed | DSB | 1 | 0.09141899 |
| TMT-A-time | DSB | 1 | 0.08905026 |
| TMT-B-time | DSB | 1 | 0 |
| PosF | PSP | 1 | 0.10824016 |
| NegF | PSP | 1 | 0.15104253 |
| DisF | PSP | 1 | 0.03058453 |
| ExcF | PSP | 1 | 0.03760063 |
| DepF | PSP | 1 | 0 |
| YMRS | PSP | 1 | 0.09327502 |
| Anxiety | PSP | 1 | 0.13606588 |
| Depression | PSP | 1 | 0 |
| Insomnia | PSP | 1 | 0 |
| Somatic | PSP | 1 | 0 |
| Logical memory immediate | PSP | 1 | 0 |
| Logical memory delayed | PSP | 1 | 0 |
| TMT-A-time | PSP | 1 | 0 |
| TMT-B-time | PSP | 1 | 0 |
| DSB | PSP | 1 | 0.11602969 |
| PosF | Onsetage | 1 | 0 |
| NegF | Onsetage | 1 | 0 |
| DisF | Onsetage | 1 | 0 |
| ExcF | Onsetage | 1 | 0 |
| DepF | Onsetage | 1 | 0.06544798 |
| YMRS | Onsetage | 1 | 0.09855509 |
| Anxiety | Onsetage | 1 | 0 |
| Depression | Onsetage | 1 | 0.17976134 |
| Insomnia | Onsetage | 1 | 0 |
| Somatic | Onsetage | 1 | 0 |
| Logical memory immediately | Onsetage | 1 | 0 |
| Logical memory delayed | Onsetage | 1 | 0 |
| TMT-A-time | Onsetage | 1 | 0 |
| TMT-B-time | Onsetage | 1 | 0 |
| DSB | Onsetage | 1 | 0 |
| PSP | Onsetage | 1 | 0 |

Note: PANSS, the Positive and Negative Syndrome Scale; PosF, positive factor; NegF, negative factor; DisF, disorganized factor; ExcF, excited factor; DepF, depressive factor;HAMD, Hamilton depression scale; YMRS, Young mania rating scale; TMT, trail making test; DSB, digital symbol substitution; PSP, personal and social performance.

**Supplementary Figures**

**Supplementary Figure 1 Network analysis of the validation cohort at the dimensional level**

**
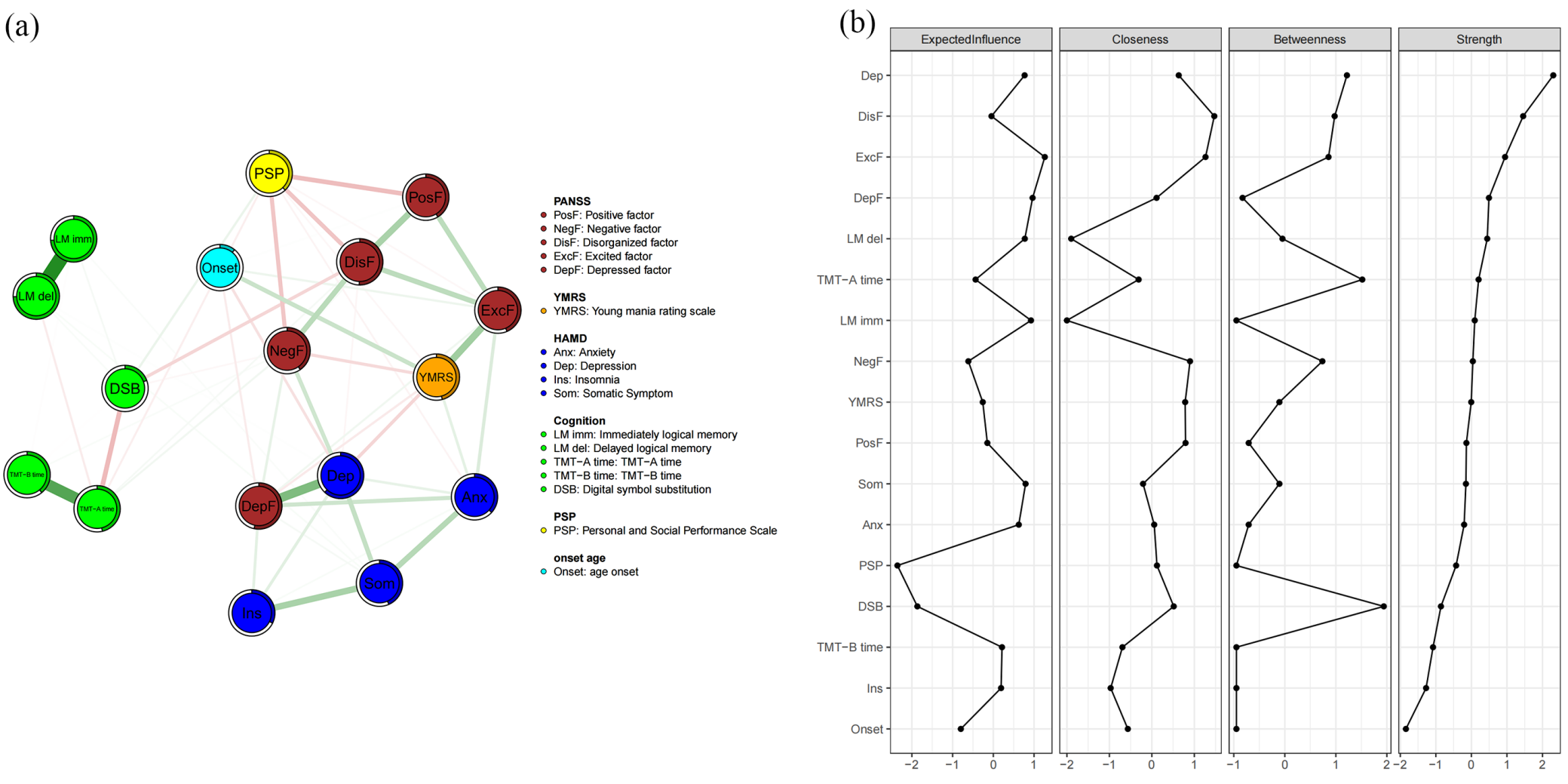
**

**Supplementary Figure 1** The estimated regularized network structure of psychotic symptoms dimensions, mood symptom dimensions, cognitive dimensions, personal functioning and duration of illness in the trans-diagnostic sample (left, a) and the centrality indices of nodes in the network (right, b). The value of each edge represents the strength of the correlations. The green edges (for the online version) or positive edge values (for the print version) indicate positive partial correlations, while the red edges (for the online version) or negative edge values (for the print version) indicate negative partial correlations. Thicker lines represent stronger connections. The ring around each node represents its predictability values. Centrality indices are shown as standardized z scores.

Note: PosF, positive factor; NegF, negative factor; DisF, disorganized factor; ExcF, excited factor; DepF, depressive factor; Anx, anxiety; Dep, depression; Som, somatic symptom; Ins, insomnia;YMRS, Young’s mania rating scale; LM, logical memory; TMT, trial making task; DSB, digital number substitution; PSP, personal and social performace scale.

**Supplementary Figure 2 Network analysis of the validation cohort at the item level**

**
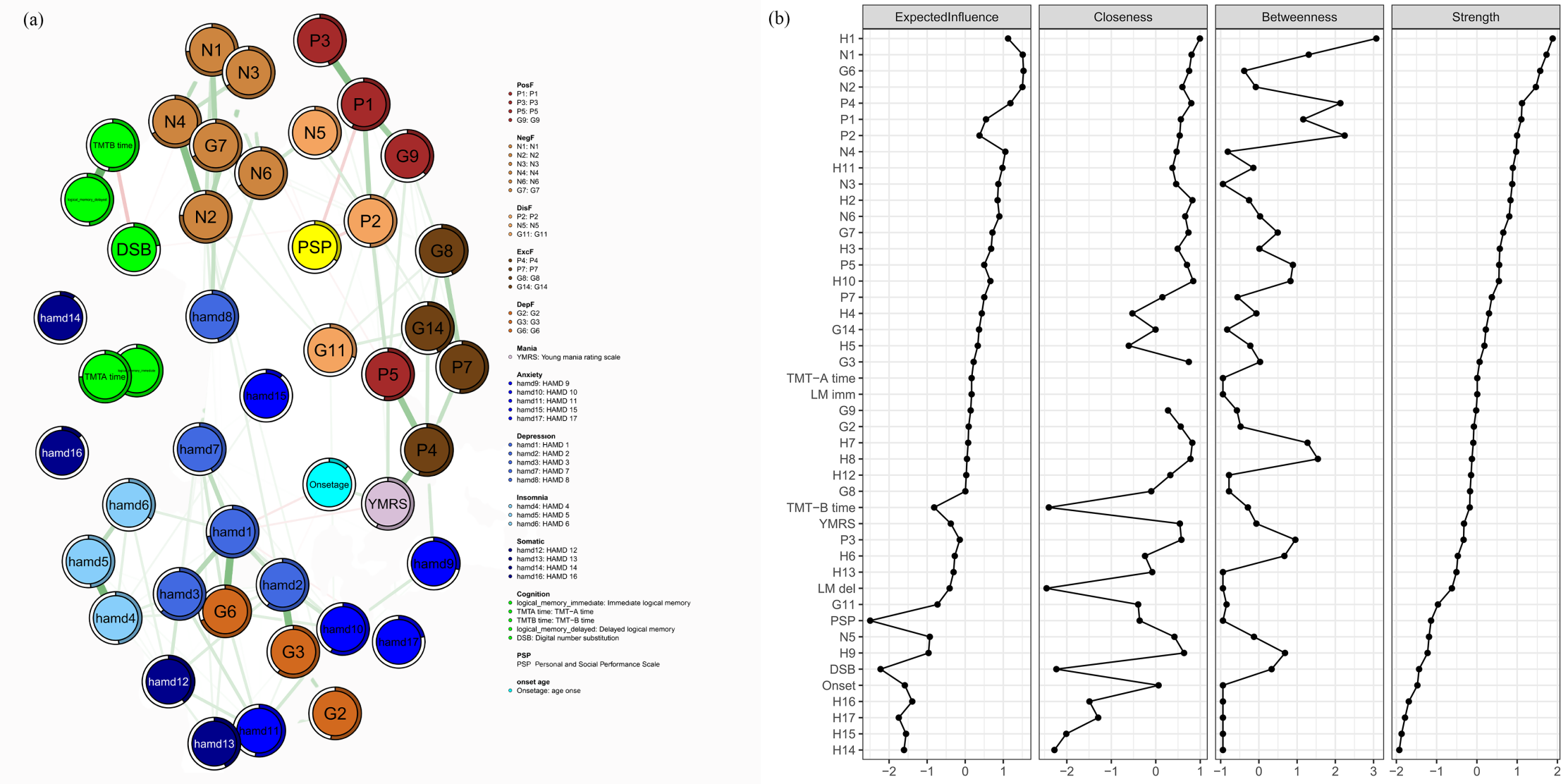
**

**Supplementary Figure 2** The estimated regularized network structure of psychotic symptoms (item level), manic symptom, depressive symptom (item level), cognitive function, personal functioning and duration of illness in the transdiagnostic sample (left) and the centrality indices of nodes in the network (right). The value of each edge represents the strength of the correlations. The green edges (for the online version) or positive edge values (for the print version) indicate positive partial correlations, while the red edges (for the online version) or negative edge values (for the print version) indicate negative partial correlations. Thicker lines represent stronger connections. The ring around each node represents its predictability values. Centrality indices are shown as standardized z scores.

Note:P, positive symptom; N, negative symptom; G, general psychopathology; H, Hamilton depression scale;YMRS, Young’s mania rating scale; LM, logical memory; TMT, trial making task; DSB, digital number substitution; PSP, personal and social performace scale.

**Supplementary Figure 3 Dimensional level directed acyclic graph in the validation cohort**

**
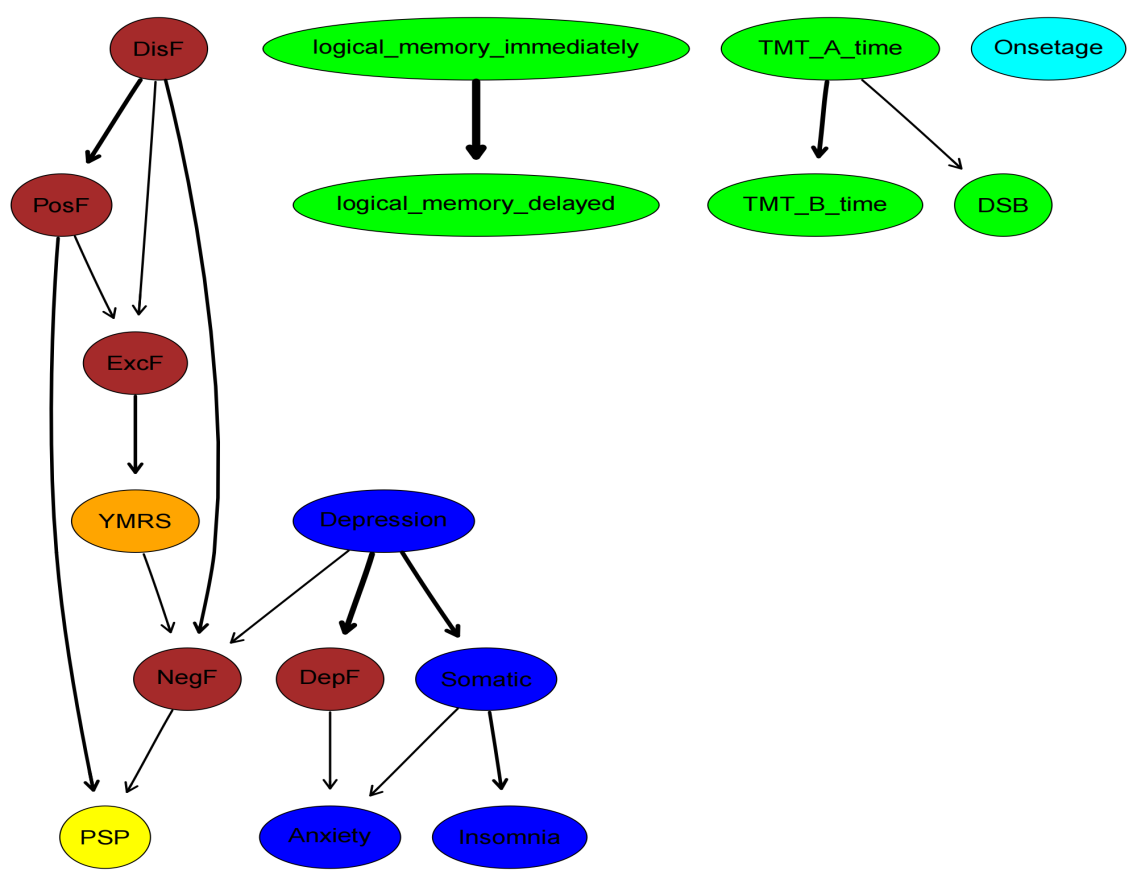
**

**Supplementary Figure 3** A consensus Bayesian network (directed acyclic graph, DAG) depicting the associations among psychopathology, cognitive function, personal functioning and illness duration at dimension-level in the validation cohort. Arrowheads show possibly predictive direction, with thicker lines for higher BIC values.

Note: PosF, positive factor; NegF, negative factor; DisF, disorganized factor; ExcF, excited factor; DepF, depressive factor; Anx, anxiety; Dep, depression; Som, somatic symptom; Ins, insomnia;YMRS, Young’s mania rating scale; LM, logical memory; TMT, trial making task; DSB, digital number substitution; PSP, personal and social performance.

**Supplementary Figure 4 Item level directed acyclic graph in the validation cohort**


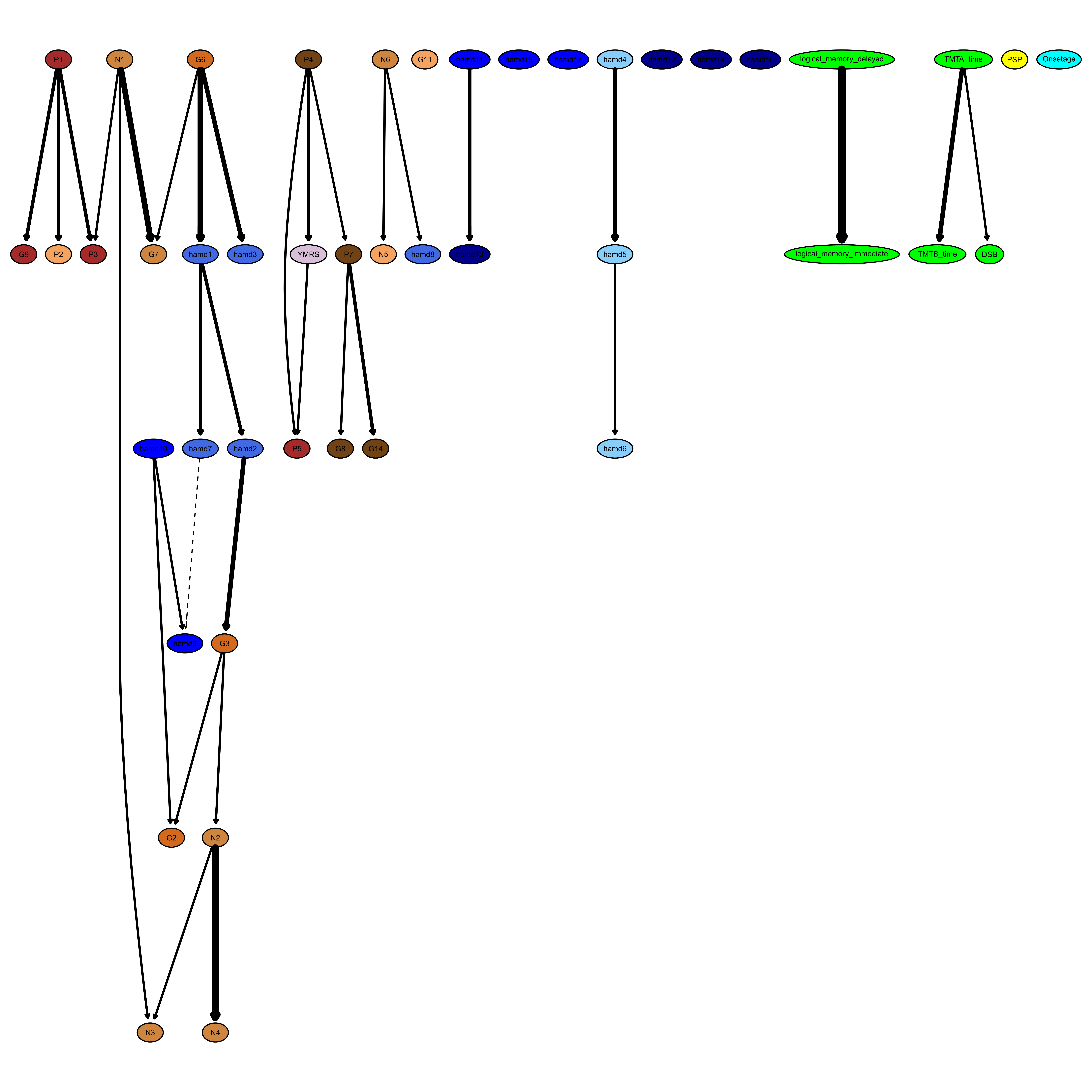


**Supplementary Figure 4** A consensus Bayesian network (directed acyclic graph, DAG) depicting the associations among psychopathology, cognitive function, personal functioning and illness duration at item-level in the validation cohort. Arrowheads show possibly predictive direction, with thicker lines for higher BIC values.

Note:P, positive symptom; N, negative symptom;G, general psychopathology; H, Hamilton depression scale;YMRS, Young’s mania rating scale; LM, logical memory; TMT, trial making task; DSB, digital number substitution; PSP, personal and social performance.
